# Single-molecule long-read sequencing reveals the chromatin basis of gene expression

**DOI:** 10.1101/gr.251116.119

**Published:** 2019-08

**Authors:** Yunhao Wang, Anqi Wang, Zujun Liu, Andrew L. Thurman, Linda S. Powers, Meng Zou, Yue Zhao, Adam Hefel, Yunyi Li, Joseph Zabner, Kin Fai Au

**Affiliations:** 1 Department of Biomedical Informatics, The Ohio State University, Columbus, Ohio 43210, USA;; 2 Department of Internal Medicine, University of Iowa, Iowa City, Iowa 52242, USA;; 3 Department of Biostatistics, University of Iowa, Iowa City, Iowa 52242, USA

## Abstract

Genome-wide chromatin accessibility and nucleosome occupancy profiles have been widely investigated, while the long-range dynamics remain poorly studied at the single-cell level. Here, we present a new experimental approach, methyltransferase treatment followed by single-molecule long-read sequencing (MeSMLR-seq), for long-range mapping of nucleosomes and chromatin accessibility at single DNA molecules and thus achieve comprehensive-coverage characterization of the corresponding heterogeneity. MeSMLR-seq offers direct measurements of both nucleosome-occupied and nucleosome-evicted regions on a single DNA molecule, which is challenging for many existing methods. We applied MeSMLR-seq to haploid yeast, where single DNA molecules represent single cells, and thus we could investigate the combinatorics of many (up to 356) nucleosomes at long range in single cells. We illustrated the differential organization principles of nucleosomes surrounding the transcription start site for silent and actively transcribed genes, at the single-cell level and in the long-range scale. The heterogeneous patterns of chromatin status spanning multiple genes were phased. Together with single-cell RNA-seq data, we quantitatively revealed how chromatin accessibility correlated with gene transcription positively in a highly heterogeneous scenario. Moreover, we quantified the openness of promoters and investigated the coupled chromatin changes of adjacent genes at single DNA molecules during transcription reprogramming. In addition, we revealed the coupled changes of chromatin accessibility for two neighboring glucose transporter genes in response to changes in glucose concentration.

In eukaryotic organisms, cells are faced with genetic information storage and packaging problems. As the carrier of genetic information, instead of folding into a disorganized yarn ball, DNA strands wrap around thousands of protein cores like “beads on a string.” As the fundamental unit of chromatin, a nucleosome consists of ∼147 bp of DNA wrapping around a histone octamer composed of four core histones (H2A, H2B, H3, and H4) (Luger et al. 1997). Nucleosomes are connected by stretches of “linker DNA.” Dynamic packaging of nucleosomes results in two different chromatin accessibility statuses: open (accessible and active genomic regions with sparse nucleosome occupancy); and closed (inaccessible and inactive genomic regions with dense nucleosome occupancy). Positioning of nucleosomes and dynamic changes of chromatin status play important regulatory roles in DNA-templated processes such as transcription, DNA replication and repair (Bell et al. 2011).

Current genome-wide methods of nucleosome occupancy and/or chromatin accessibility mapping are mainly based on three types of assays followed by short-read sequencing technologies: (1) the nucleosome's protection of nucleosomal DNA sequences from endogenous and exogenous enzymes (e.g., MNase-seq, DNase-seq, ATAC-seq, NOMe-seq, and MPE-seq) (Schones et al. 2008; Song and Crawford 2010; Cui and Zhao 2012; Kelly et al. 2012; Buenrostro et al. 2015a; Ishii et al. 2015); (2) chromatin immunoprecipitation using a specific histone antibody (e.g., ChIP-seq with H3) (Wal and Pugh 2012); and (3) solubility differences between nucleosomal DNA and naked linker DNA (e.g., FAIRE-seq) (Bianco et al. 2015). In particular, NOMe-seq treats target samples with exogenous methyltransferase to detect nucleosome occupancy and chromatin accessibility: The nucleosome protects nucleosomal DNA from being methylated by exogenous methyltransferase, while cytosines in naked linker DNA sequences are methylated to 5-methylcytosine (5mC) (Kelly et al. 2012). The following bisulfite sequencing identifies this methylation profile, as bisulfite can convert unmethylated cytosine to uracil, which discriminates 5mC from unmethylated cytosine.

These methods can map averaged patterns of nucleosome occupancy and chromatin accessibility in a population of cells, failing in precise identification at the single-cell level. Although the single-cell versions of these methods have been recently developed (Small et al. 2014; Buenrostro et al. 2015b; Jin et al. 2015; Pott 2017; Clark et al. 2018; Lai et al. 2018; Li et al. 2018), the corresponding sparse sequencing coverage and short read length lack information for addressing complex long-range chromatin status and nucleosome occupancy. Therefore, the heterogeneity of nucleosome occupancy and chromatin accessibility is rarely studied. Moreover, it is even more challenging to define nucleosome occupancy patterns and dynamics and chromatin accessibility at single DNA molecules, so it is hard to detect subtle but meaningful differences between seemingly identical cells. This is a critical gap in understanding the mechanism of how nucleosomes assemble, disassemble, and slide. In addition, in contrast to the well-solved phasing problems (e.g., exon-splicing and allele-specific DNA methylation), the phased status of nucleosomes and chromatin accessibility at single DNA molecules remains incomplete (Kuleshov et al. 2014; Tilgner et al. 2018).

The emerging single-molecule long-read sequencing technology (i.e., Oxford Nanopore Technologies, ONT) provides unique data features that are capable of filling the gap: (1) 5mC can be directly detected at the single-base resolution at the single-molecule level based on ONT electrolytic current signal dynamics without bisulfite conversion (Rand et al. 2017; Simpson et al. 2017); (2) unlike the other sequencing platforms (such as Sanger sequencing and second-generation sequencing [e.g., Illumina]), PCR amplification is not required for ONT sequencing, so each ONT read can reveal the genomic events at the single-molecule level; (3) ONT reads are ultralong (up to 2.3 Mb) (Payne et al. 2018) so that they can cover combinatorics of many nucleosomes and different chromatin statuses spanning multiple genomic elements. Leveraging the informative ONT sequencing technology, we developed an experimental approach, methyltransferase treatment followed by ONT single-molecule long-read sequencing (MeSMLR-seq) and the corresponding bioinformatics method, to investigate heterogeneous and dynamic insight into long-range chromatin status and nucleosomes. Instead of bisulfite conversion (with PCR amplification) and short-read sequencing, the footprint of exogenous 5mCs from GpC-specific methyltransferase treatment is detected at single DNA molecules (without any PCR amplification) by ONT sequencing in the MeSMLR-seq protocol and is next used to detect nucleosome occupancy and chromatin accessibility computationally.

We applied MeSMLR-seq to haploid *Saccharomyces cerevisiae* cells, where single DNA molecules represent single cells, so it allows the “one-to-one” link between sequencing read (i.e., sequencing molecule) and haploid cell. Thus, each single MeSMLR-seq read can be used to mimic a single cell in a given genomic region, and the heterogeneity can be investigated without single-cell sequencing. With the unique output of MeSMLR-seq, we revealed the chromatin basis of gene transcription.

## Results

### Overview of MeSMLR-seq

**Figure 1.**
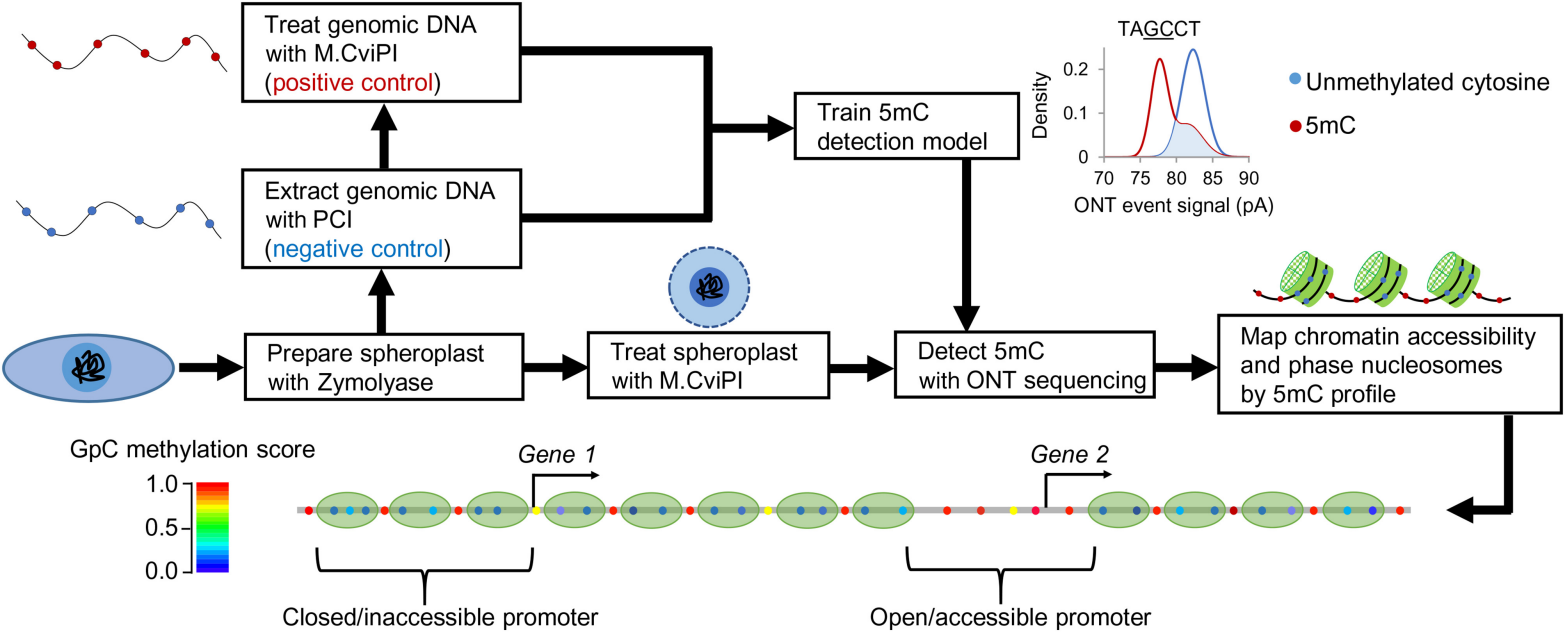
Overview of MeSMLR-seq. Experimental approach (methyltransferase treatment plus ONT sequencing) in yeast and the corresponding bioinformatics analyses (5mC detection, chromatin accessibility mapping, and nucleosome phasing).

In brief, the experimental approach MeSMLR-seq contains two main steps: (1) methyltransferase (M.CviPI) treatment to convert cytosine to 5mC at GpC sites at naked linker DNA and open chromatin; and (2) ONT sequencing to detect the 5mC profile that is subsequently used to identify nucleosome occupancy and chromatin accessibility (Fig. 1). The first step has been shown feasible at both the bulk-cell and single-cell level by NOMe-seq and other previous studies (Small et al. 2014; Pott 2017; Clark et al. 2018; Li et al. 2018). In addition, ONT has been reported to detect 5mC at CpG sites (Rand et al. 2017; Simpson et al. 2017), based on which an in-house tool (named NP-SMLR) (see Supplemental Code) was developed to map the 5mC profile at GpC sites for MeSMLR-seq data.

In the proof-of-concept application of MeSMLR-seq to a haploid *Saccharomyces cerevisiae* (BY4741 strain), an additional step was applied to digest the cell wall that serves as a barrier against methyltransferase treatment of genomic DNA: Yeast cells were treated with Zymolyase to generate spheroplasts (Fig. 1). After the subsequent methyltransferase treatment, extracted genomic DNA without any PCR amplification was directly submitted to library preparation and ONT sequencing. The genomic DNA that underwent in vivo spheroplast methylation was referred to as the target sample of MeSMLR-seq. In addition, we prepared negative control and positive control samples as training data for 5mC detection (see the next section “Detection and phasing of nucleosome occupancy at single DNA molecules”). Native genomic DNA extracted from yeast without M.CviPI treatment was used as a negative control (all cytosines at GpC sites were unmethylated) since there is no endogenous 5mC on the yeast genome, as previously reported (Capuano et al. 2014). Genomic DNA treated with M.CviPI (without spheroplast methylation) was used as a positive control (all cytosines at GpC sites were converted to 5mCs).

As the efficiency of M.CviPI methylation served a critical role in the whole protocol, it was evaluated at selected genomic regions by bisulfite sequencing as previously described (Small et al. 2014). The methylation efficiency of the positive control sample was 99.37%, and 13 single colonies of the selected region from target sample were all successfully methylated, indicating high methylation efficiency.

Using the ONT GridION platform with R9.4.1 chemistry, we sequenced one flow cell per sample and generated 0.9 million (positive control), 1.2 million (negative control), and 1.3 million (on average for six target samples) reads (i.e., sequencing molecules) separately, which were uniquely aligned to the yeast genome (Supplemental Table S1). The longest sequencing molecule was 63.1 kb. In particular, from the target sample where yeast was grown in rich media (1% yeast extract, 2% peptone, and 2% glucose), we generated 1.4 million sequencing molecules with a median length of 7.2 kb, covering 821× of the yeast genome.

### Detection and phasing of nucleosome occupancy at single DNA molecules

**Figure 2.**
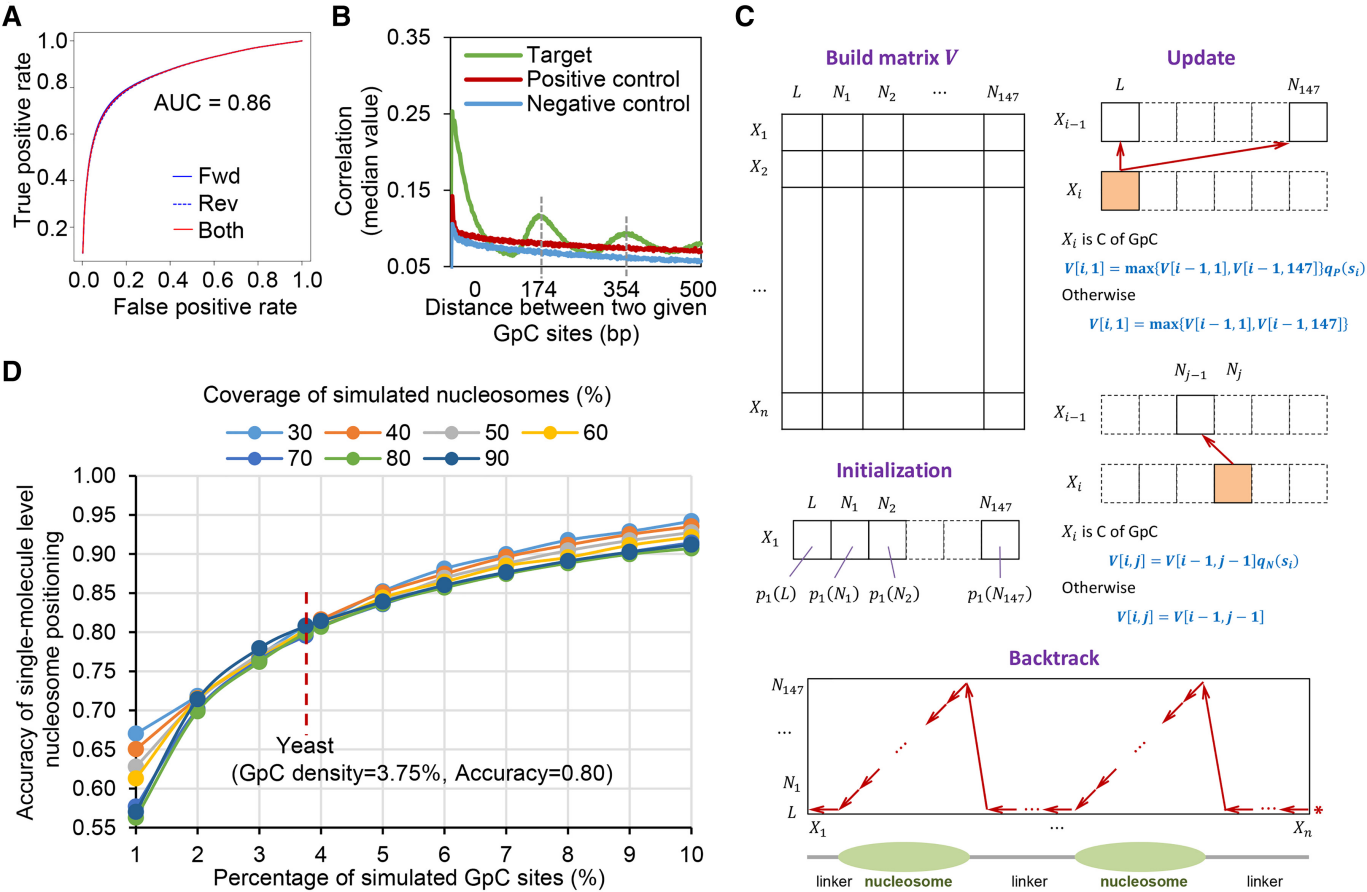
5mC detection and nucleosome occupancy detection by MeSMLR-seq data. (*A*) ROC curve of 5mC detection on GpC sites. The molecules that were aligned to forward (fwd) and reverse (rev) genomic strands were analyzed separately. (*B*) Correlation coefficients between methylation scores of mutually paired GpC sites from the same molecules with respect to their corresponding distances. (*C*) Dynamic programming algorithm for nucleosome occupancy detection (NP-SMLR). A matrix regarding the nucleotide sequence (row) and nucleosomal statuses (column) is made, followed by initialization, iterative update for entries, and backtrack search for optimal path (see Methods for details). (*D*) Accuracy of nucleosome occupancy detection under different nucleosome coverage and GpC frequencies.

We first identified 5mC methylation status for every GpC site on each DNA molecule based on the ONT sequencing current signal (referred to as the event level). Since the previous studies (Rand et al. 2017; Simpson et al. 2017) showed the event level depended on the context sequence (e.g., 6-mer), our positive and negative control data were used to train signal distributions for each 6-mer containing target GpC dinucleotides under the occasions of methylation and unmethylation. The event levels of a given 6-mer from the target sample were compared with the corresponding trained distributions to obtain a posterior of methylation for every GpC site on each molecule, which we denoted as the methylation score (Supplemental Fig. S1A). There was no obvious bias of 5mC methylation calling between the molecules that were aligned to forward and reverse strands, and the areas under the receiver operating characteristic curve (AUC) were both 0.86 (Fig. 2A). Correlation analysis of methylation status of paired GpC sites at single molecules showed a pattern with a periodic distance of 170–180 bp, which was the same as the length of nucleosomal DNA (147 bp) plus regular linker DNA (20–30 bp) (Fig. 2B). Therefore, we can identify nucleosome occupancy at single molecules from the methylation profiles by developing the bioinformatics method **N**ucleosome **P**ositioning detection by **S**ingle-**M**olecule **L**ong-**R**ead sequencing (**NP-SMLR**) as below (see Supplemental Code).

Let *X*_1_ *X*_2_…*X*_*l*_ be a molecule, where *X*_*i*_ is the *i*-th base. Denote *s*_*i*_ as the methylation score of *X*_*i*_, if *X*_*i*_ is the cytosine of the GpC dinucleotide. Suppose that the methylation scores of all GpC sites are independent. Nucleosome occupancy detection refers to finding a path *π* = *π*_1_*π*_2_…*π*_*l*_ that maximizes the likelihood of signals:

$$\pi^{*}=\underset{\pi }{\mathrm{argmax}}\overset{n}{\underset{t=1}{\prod }}\mathrm{Pr}(s_{i_{t}}|\pi_{i_{t}}).$$

*π*_*i*_ takes the value from {*L*, *N*_1_, *N*_2_, …, *N*_147_}. *L* represents the linker region; *N*_*m*_ represents the *m*-th base within a nucleosome; *i*_1_, *i*_2_, …, *i*_*n*_ are the positions of cytosines that belong to GpC dinucleotides. The elements of path *π* are restricted such that: (1) *N*_*m*_ is followed by *N*_*m*+1_ (1 ≤ *m* ≤ 146); (2) *N*_147_ is followed by *L*; and (3) *L* is followed by *L* or *N*_1_. The problem is essentially an alignment between a sequence of nucleotides and a sequence of nucleosomal statuses. NP-SMLR adopts a dynamic programming algorithm (Needleman and Wunsch 1970) for solution: A matrix regarding the nucleotide sequence and nucleosomal statuses is made, entries are updated iteratively, and the optimal path is obtained through backtracking (Fig. 2C; Supplemental Fig. S1B).

Due to the lack of more advanced experimental technology to generate a gold standard, we evaluated the accuracy of nucleosome occupancy detection at the single-molecule level by simulation tests. The tests were performed under different settings of nucleosome coverage (proportion of bases covered by nucleosomes, ranges from 30% to 90%) and GpC frequency (ranges from 1% to 10%) (Fig. 2D). The accuracy increased with GpC frequency, while the effect of nucleosome coverage was mild. In the case of the yeast genome with 3.75% density of GpC sites, NP-SMLR was very robust to reach the accuracy of 80% regardless of different nucleosome coverages, which represented different scenarios of chromatin status (Fig. 2D). These results highlight the accuracy and robustness of MeSMLR-seq on single-molecule long-range mapping of nucleosomes.

### Performance of nucleosome occupancy detection at the bulk-cell level

**Figure 3.**
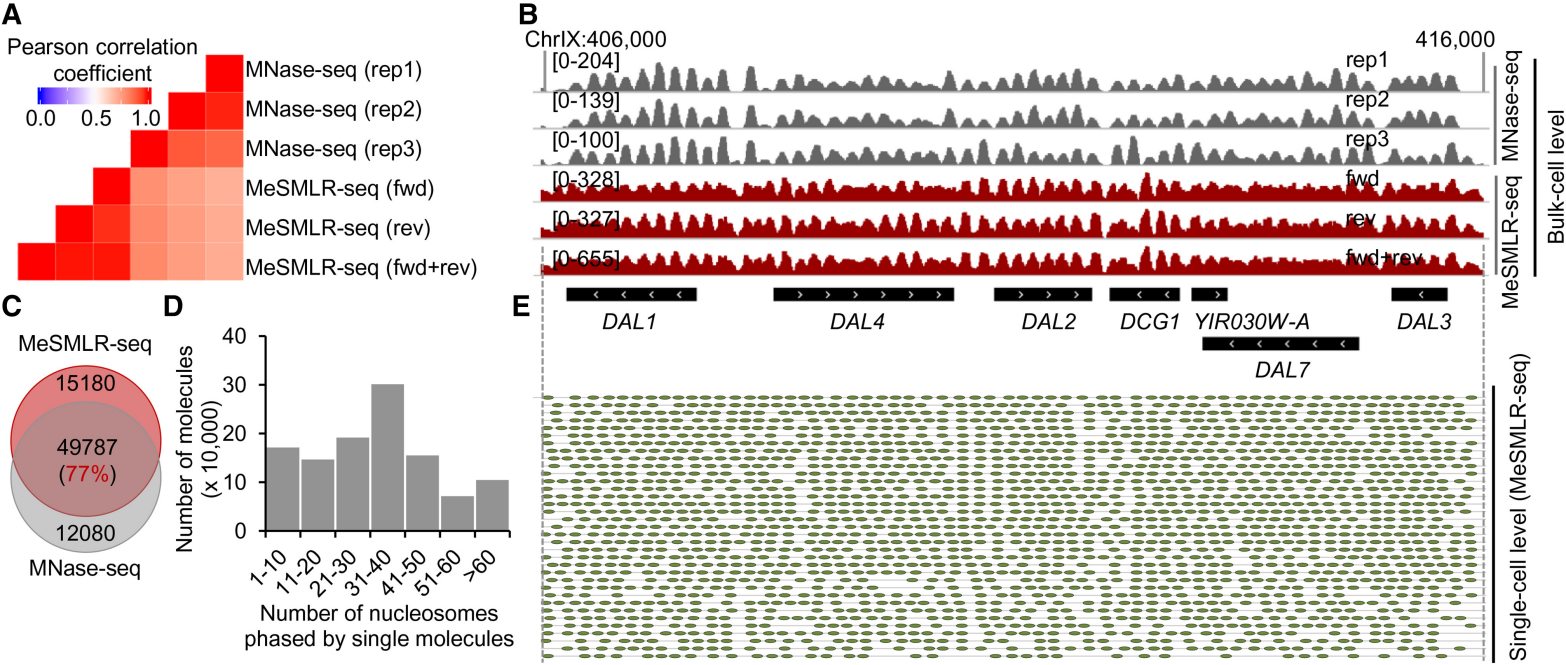
Performance evaluation of MeSMLR-seq on bulk-level nucleosome occupancy and single-molecule long-range phasing of nucleosomes. (*A*) Correlation of nucleosome occupancy profiles generated by MeSMLR-seq and MNase-seq. For MeSMLR-seq, the molecules that were aligned to forward (fwd) and reverse (rev) genomic strands were analyzed separately. (*B*) Nucleosome occupancy profiles at the bulk-cell level provided by MeSMLR-seq and MNase-seq. (*C*) Overlap of nucleosomes detected by MeSMLR-seq and MNase-seq at the bulk-cell level. (*D*) Number of nucleosomes phased at single sequencing molecules of MeSMLR-seq data under 2% glucose condition. (*E*) Detection and phasing of nucleosomes at the single-molecule level by NP-SMLR. Each gray line represents a molecule. Green oval represents nucleosome.

In terms of nucleosome occupancy at the bulk-cell level, MeSMLR-seq provided consistent and comparable results with the widely used method MNase-seq (Supplemental Methods; Hughes and Rando 2014; Weiner et al. 2015). The averaged Pearson's correlation coefficient between three MeSMLR-seq data (forwardly, reversely aligned molecules, and their combination) and three MNase-seq replicates was 0.75 (Fig. 3A). The 77% of nucleosomes called by MeSMLR-seq were also detected by MNase-seq (Fig. 3C). For an example of the *DAL* (degradation of allantoin) gene cluster, the nucleosome peaks called by MeSMLR-seq and MNase-seq were generally well aligned (Fig. 3B). In the long-range scale, single MeSMLR-seq reads can phase a number of nucleosomes (the median number was 37 and the maximal number was 356 in our data), so that it captures the dynamics and heterogeneity of nucleosome occupancy among DNA molecules (Fig. 3D; Supplemental Table S2). For instance, 35–61 nucleosomes (median number 58) were phased at the single molecules covering the *DAL* gene cluster across a 10-kb genomic region (Fig. 3E), which illustrated large-range variation as well as local subtle differences of nucleosome occupancy.

### Direct long-range evidence of differential nucleosome organization

**Figure 4.**
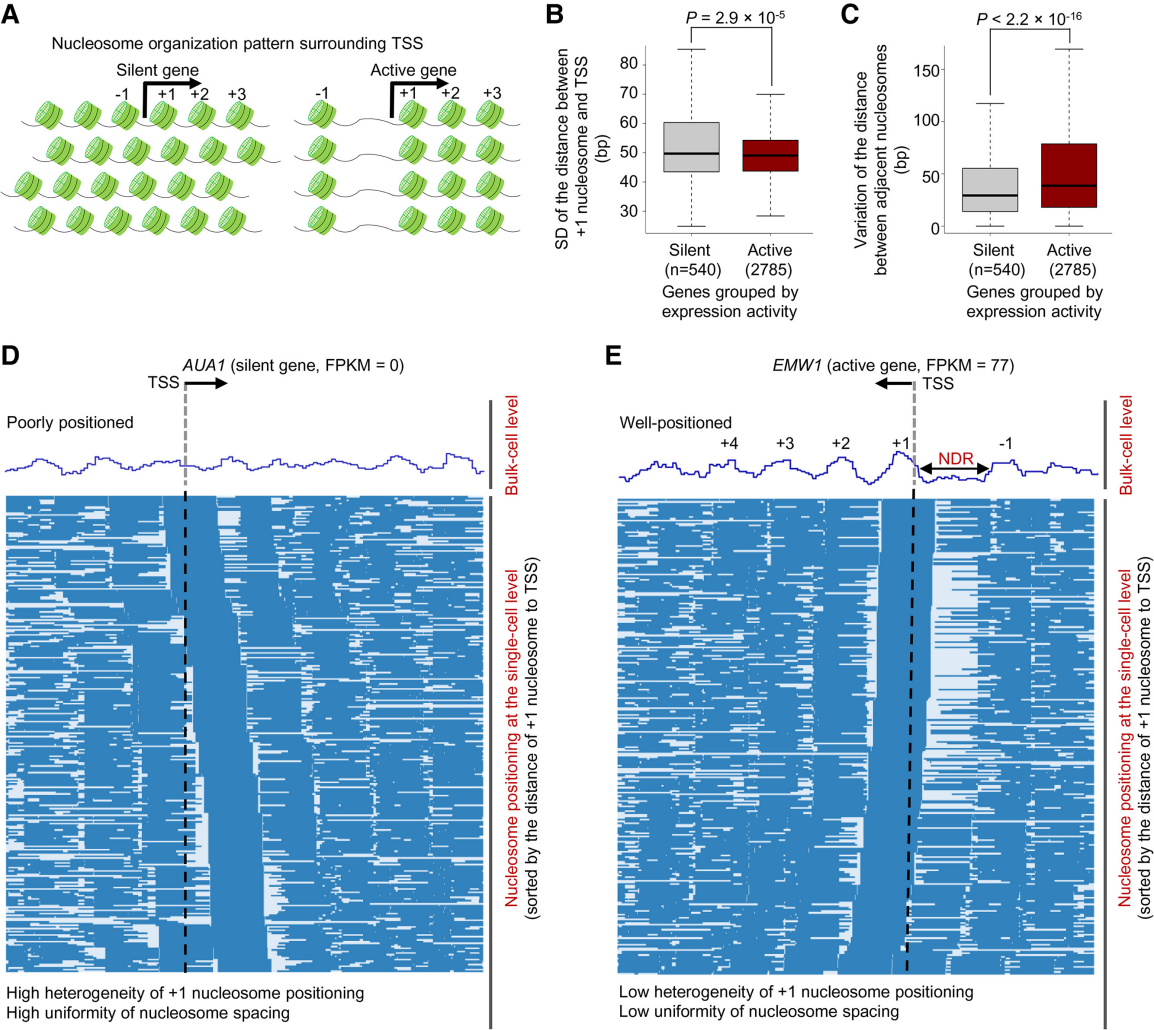
Differential nucleosome organization principles for silent and active genes. (*A*) Previous studies revealed nucleosome organization patterns surrounding the TSS of silent (*left*) and active (*right*) genes (Lai et al. 2018). Nucleosome positioning in promoter regions of silent genes showed large variation among cells but was highly uniformly spaced within each cell. In contrast, nucleosome positioning surrounding the TSS of active genes showed little variation among cells but relatively nonuniform spacing within each cell. (*B*) Heterogeneity of nucleosome positioning for silent (FPKM = 0) and active (FPKM > 50) genes. The heterogeneity of nucleosome positioning was measured by the standard deviation (SD) of the distances between +1 nucleosomes and the TSS. The *P*-value was calculated by the Wilcoxon rank-sum test. (*C*) Uniformity of nucleosome spacing for silent (FPKM = 0) and active (FPKM > 50) genes. See Methods for the definition of uniformity. The *P*-value was calculated by the Wilcoxon rank-sum test. (*D*) Long-range nucleosome positioning patterns for the silent *AUA1* across different cells. Each row represents a cell, and nucleosome is labeled as blue bar. (*E*) Long-range nucleosome positioning patterns for the actively transcribed gene *EMW1* across different cells.

A few single-cell epigenome sequencing approaches have revealed the heterogeneity of chromatin status and nucleosome positioning within a cell population (Small et al. 2014; Buenrostro et al. 2015b; Jin et al. 2015; Pott 2017; Clark et al. 2018; Lai et al. 2018; Li et al. 2018). Recently, Lai et al. reported the differential nucleosome organization principles for silent and active genes using single-cell MNase-seq (Fig. 4A; Lai et al. 2018). However, these studies lacked a long-scale nucleosome occupancy scene at the single-cell resolution due to short sequencing length and sparse data coverage within single cells. As shown above, MeSMLR-seq can determine the heterogeneous long-range phasing of nucleosomes, so we can investigate nucleosome organization logic in a comprehensive way (Fig. 3E).

We focused on the nucleosome organization surrounding transcription start sites (TSSs), which play an important role in transcription regulation (Voss and Hager 2014). For each gene, we measured the heterogeneity of nucleosome positioning by the standard deviation of the distances between +1 nucleosome and the TSS over all single cells. Compared to active genes, silent genes showed a larger heterogeneity of nucleosome positioning among different cells (Fig. 4B; Supplemental Fig. S2A). Next, we evaluated the uniformity of nucleosome spacing within single cells by the variation of the distance between adjacent nucleosomes. In contrast to active genes, the nucleosomes surrounding the TSS of silent genes were more uniformly spaced (Fig. 4C; Supplemental Fig. S2B). For instance, at the bulk-cell level, nucleosomes surrounding the TSS of the lowly expressed gene *AUA1* (FPKM = 0) were poorly positioned (Fig. 4D), while there were well-positioned nucleosomes (including −1, +1, +2, +3, and +4 nucleosomes) surrounding the TSS of the active gene *EMW1* (FPKM = 77) and a pronounced nucleosome-depletion region (NDR) upstream of the TSS (Fig. 4E). At the single-cell level, the positioning of +1 nucleosome of *AUA1* had a continuous shift pattern across different cells, whereas it was relatively steady for *EMW1* (Fig. 4D,E). Compared with *EMW1*, the distances between +1 nucleosomes and the TSS for *AUA1* were more approximate to a uniform distribution (Supplemental Fig. S3A,B), which represented the ideal occasion for a continuous shift pattern. In addition, the spacing of nucleosomes surrounding the TSS of *AUA1* was relatively uniform within single cells (Fig. 4D; Supplemental Fig. S3C), while there was a pronounced NDR upstream of the TSS of *EMW1*, which disrupted the uniformity of nucleosome spacing (Fig. 4E; Supplemental Fig. S3D). MeSMLR-seq resolves these differential nucleosome organization principles with direct and convincing evidence at a long-range scale from single molecules/cells that are hard to obtain by the bulk-cell and short-read sequencing approaches.

### Single-molecule long-range measurement of chromatin accessibility

**Figure 5.**
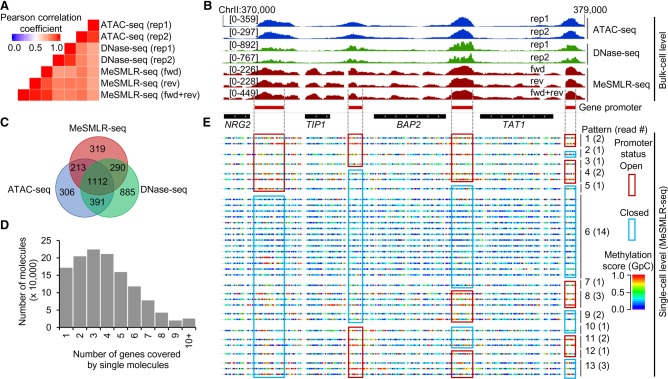
Performance evaluation of MeSMLR-seq on bulk-level chromatin accessibility mapping, and single-molecule long-range mapping of chromatin accessibility. (*A*) Correlation of chromatin accessibility profiles generated by MeSMLR-seq, ATAC-seq, and DNase-seq. (*B*) Chromatin accessibility profiles at the bulk-cell level provided by MeSMLR-seq, ATAC-seq, and DNase-seq. (*C*) Overlap of the significantly accessible regions (peaks) called by MeSMLR-seq, ATAC-seq, and DNase-seq. (*D*) Number of genes covered by single sequencing molecules of MeSMLR-seq data under 2% glucose condition. (*E*) Single-molecule long-range mapping of chromatin accessibility by MeSMLR-seq. Each line represents a molecule. GpC site is labeled as a rainbow-color dot, with methylation score from 0 (blue) to 1.0 (red). Thirteen combinatorial patterns of the promoter status of four genes are shown with different numbers of supporting sequencing molecules/cells. A promoter was defined as “open” (highlighted by red box) if the methylation scores of the including GpC sites had a median value greater than 0.5, and “closed” (highlighted by blue box) otherwise.

Based on the methylation profiles of MeSMLR-seq data, we also mapped the chromatin accessibility of the yeast genome at both the bulk-cell level and single-molecule level. To assess the performance on the bulk-cell chromatin accessibility mapping, we compared MeSMLR-seq with two widely used methods, ATAC-seq (Schep et al. 2015) and DNase-seq (Supplemental Methods; Zhong et al. 2016). The genome-wide chromatin accessibility profile revealed by MeSMLR-seq data was highly consistent with ATAC-seq (averaged Pearson's *r* = 0.80) and DNase-seq (averaged Pearson's *r* = 0.82) (Fig. 5A,B; Supplemental Fig. S4). In addition, >83% (1615/1934) of significantly accessible regions called by MeSMLR-seq were also supported by either ATAC-seq or DNase-seq (Fig. 5C). These results indicate that MeSMLR-seq provides comparable results with the existing methods on the bulk-cell level chromatin accessibility mapping.

At the single-molecule level, a MeSMLR-seq read can fully cover multiple adjacent genes (median number was 4 and maximal number was 40 in our data); therefore, we could examine the long-range chromatin accessibility at the single-molecule/-cell level (Fig. 5D; Supplemental Table S3). For example, 34 MeSMLR-seq molecules fully covered the 9-kb genomic region, Chr II: 370,000–379,000, that encompasses four genes (*NRG2*, *TIP2*, *BAP2*, and *TAT1*). Based on the 5mC footprint, we identified the chromatin status (“open” or “closed”) of the promoters for four genes on each molecule and thus defined and quantified the coupled chromatin status patterns. In total, these molecules detected 13 out of 16 (4^2^, four genes with binary status “open” or “closed”) possible combinatorial patterns of the coupled chromatin statuses of four gene promoters (Fig. 5E). For instance, four genes in Pattern 1 (supported by two molecules) all had “open” promoters, whereas the promoters of four genes were all closed in Pattern 6 (supported by 14 molecules). Therefore, MeSMLR-seq is able to analyze the coupled chromatin statuses of adjacent genes and to investigate the heterogeneity of chromatin status within a cell population, which is challenging for the existing methods.

### Heterogeneous openness of a gene promoter

**Figure 6.**
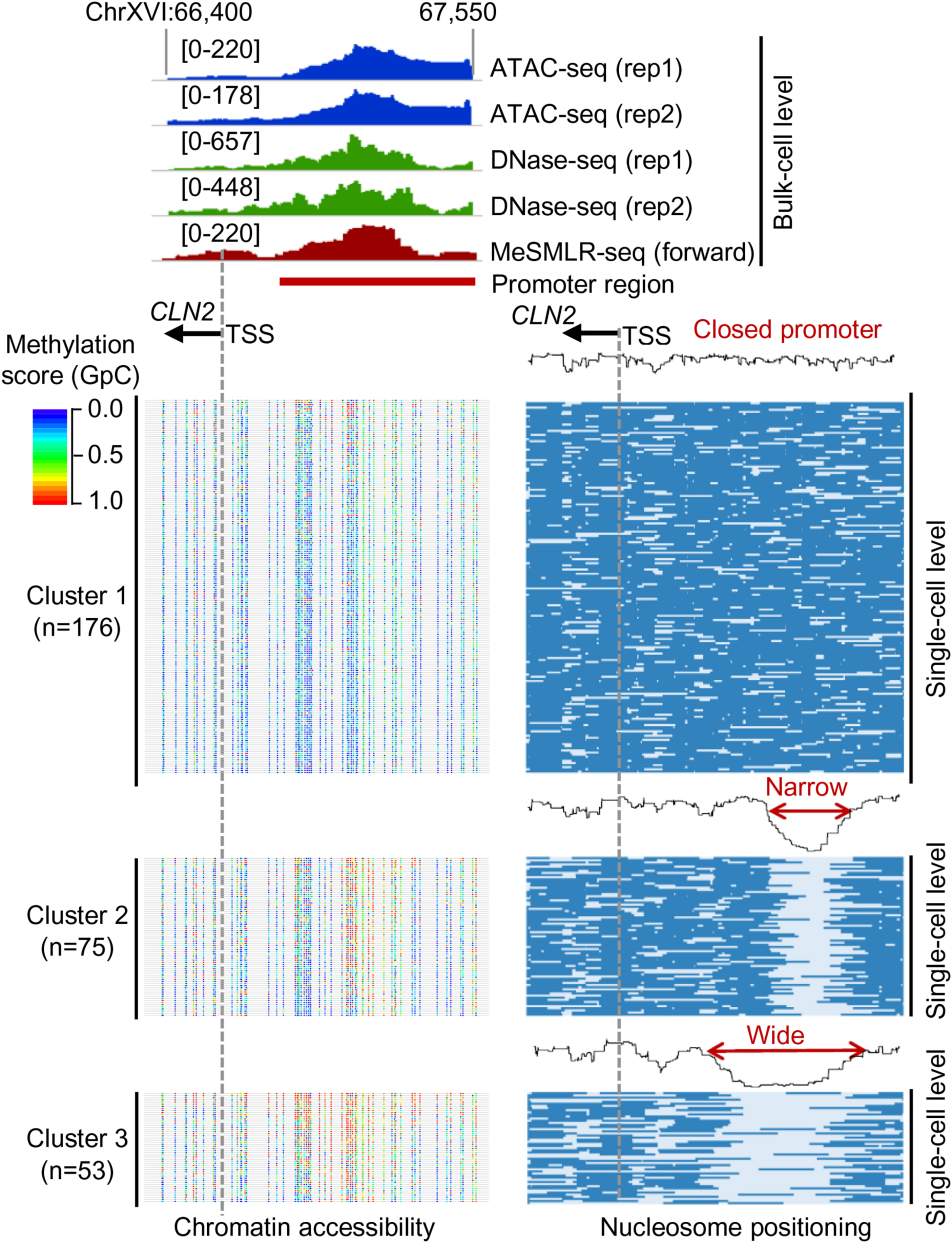
Heterogeneous promoter openness of *CLN2* in a cell population revealed by MeSMLR-seq. The bulk-level chromatin accessibility profiles (*upper* panel) were provided by ATAC-seq, DNase-seq, and MeSMLR-seq. MeSMLR-seq molecules were clustered into three groups with different promoter openness (by *k*-means clustering of the nucleosome occupancy profiles, *bottom right* panel): closed, narrow open, and wide open. Each row represents a molecule (i.e., a cell), and nucleosome is labeled as blue bar. The corresponding methylation profiles at GpC sites on each molecule are shown on the *bottom left* panel. Each line represents a molecule (i.e., a cell). GpC site is labeled as a rainbow-color dot, with methylation score from 0 (blue) to 1.0 (red).

Leveraging the single-molecule and long-range information of MeSMLR-seq data, we can discover and measure different levels of promoter openness beyond reporting a binary status. In the promoter region (Chr XVI: 66,400–67,550) of the cell cycle regulation gene *CLN2*, the bulk-level chromatin accessibility profiles generated by the existing methods and MeSMLR-seq all showed a significant openness (Fig. 6, upper panel), while it was not clear if the promoters of *CLN2* among all cells were open, or if the open regions were similar in size. Based on the single-molecule nucleosome occupancy profiles in the promoter region, 304 molecules that fully covered this region were clustered into three groups with different levels of promoter openness: closed (Cluster 1 with 176 molecules); narrowly open (Cluster 2 with 75 molecules); and widely open (Cluster 3 with 53 molecules) (Fig. 6, lower-right panel). The 5mC profiles at the molecules from three clusters also showed the difference of the widths of openness (Fig. 6, lower-left panel). This unique output of MeSMLR-seq is bringing new opportunities to perform quantitative analysis of the heterogeneous and dynamic promoter status.

In addition, the MeSMLR-seq data revealed nucleosome occupancy and chromatin accessibility profiles consistent with the previous studies based on bulk-cell short-read data (Hughes and Rando 2014) upstream of the TSS, gene body region, and binding region of several important transcriptional regulators (Supplemental Note 1; Supplemental Figs. S5, S6), as well as revealing the dynamics of chromatin status during transcription changes in response to different nutrition conditions (Supplemental Note 2; Supplemental Fig. S7).

### Quantitative relationship between gene expression and chromatin accessibility in cell populations

**Figure 7.**
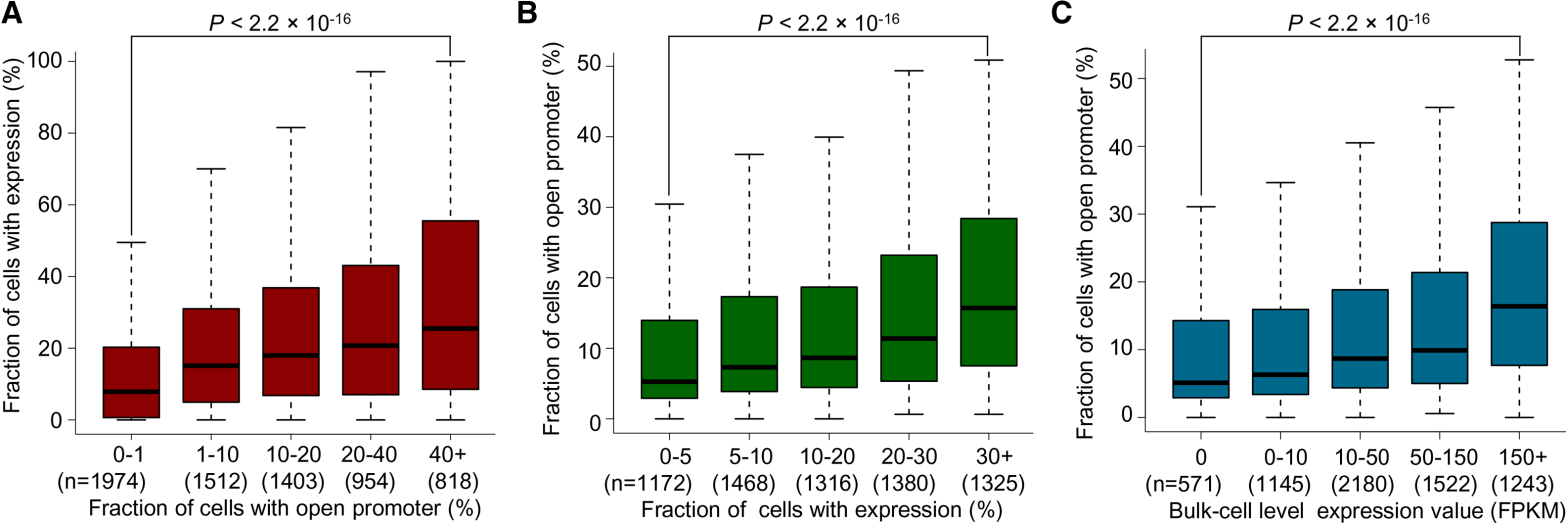
Quantitative relationship between chromatin accessibility and gene expression. (*A*,*B*) Quantitative relationship between chromatin accessibility and gene expression in a cell population. The former (*A*) was measured by the fraction of cells with an open promoter, and the latter (*B*) by the fraction of cells with expression (based on single-cell RNA-seq data). Genes were binned by one of the indices and the distribution of the other is shown. The gene was considered as “expressed” in a cell if the corresponding UMI (unique molecular identifier) count was ≥1. (*C*) Quantitative relationship between the bulk-cell gene expression and the cell population ratio of an open promoter. Genes were binned based on the bulk-cell gene expression level (RNA-seq data).

Though the analyses above showed that the promoters of the highly expressed genes over a cell population were generally more accessible than the lowly expressed genes (Supplemental Fig. S5), the quantitative relationship between promoter openness and gene transcription in a cell population remained unclear. Based on unique MeSMLR-seq data, we were able to calculate the fraction of a cell subpopulation with an open promoter of a given gene. With single-cell RNA-seq data for 2812 yeast cells generated in this study (Supplemental Methods), we also calculated the fraction of cells with expression (read count ≥1) of a given gene (referred to as expression frequency). The expression frequency within a cell population was positively correlated with the fraction of cells with an open promoter (Fig. 7A). For example, the genes with an open promoter in ≥40% cells had a significantly larger expression frequency than the ones with an open promoter in <10% cells (*P*-value <2.2 × 10^−16^, Wilcoxon rank-sum test) (Fig. 7A).

When grouping the genes based on expression frequency, we observed similar positive correlation (Fig. 7B). In addition, considering the bulk-cell expression, the highly expressed genes were present in relatively large fractions of a cell subpopulation with an open promoter in comparison to the lowly expressed ones (*P*-value <2.2 × 10^−16^, Wilcoxon rank-sum test) (Fig. 7C). These results suggest that chromatin accessibility of a promoter at the single-molecule/-cell level detected by MeSMLR-seq data can contribute to the prediction of gene expression level and frequency in a cell population.

### Coupled chromatin accessibility changes of adjacent genes during transcription reprogramming

**Figure 8.**
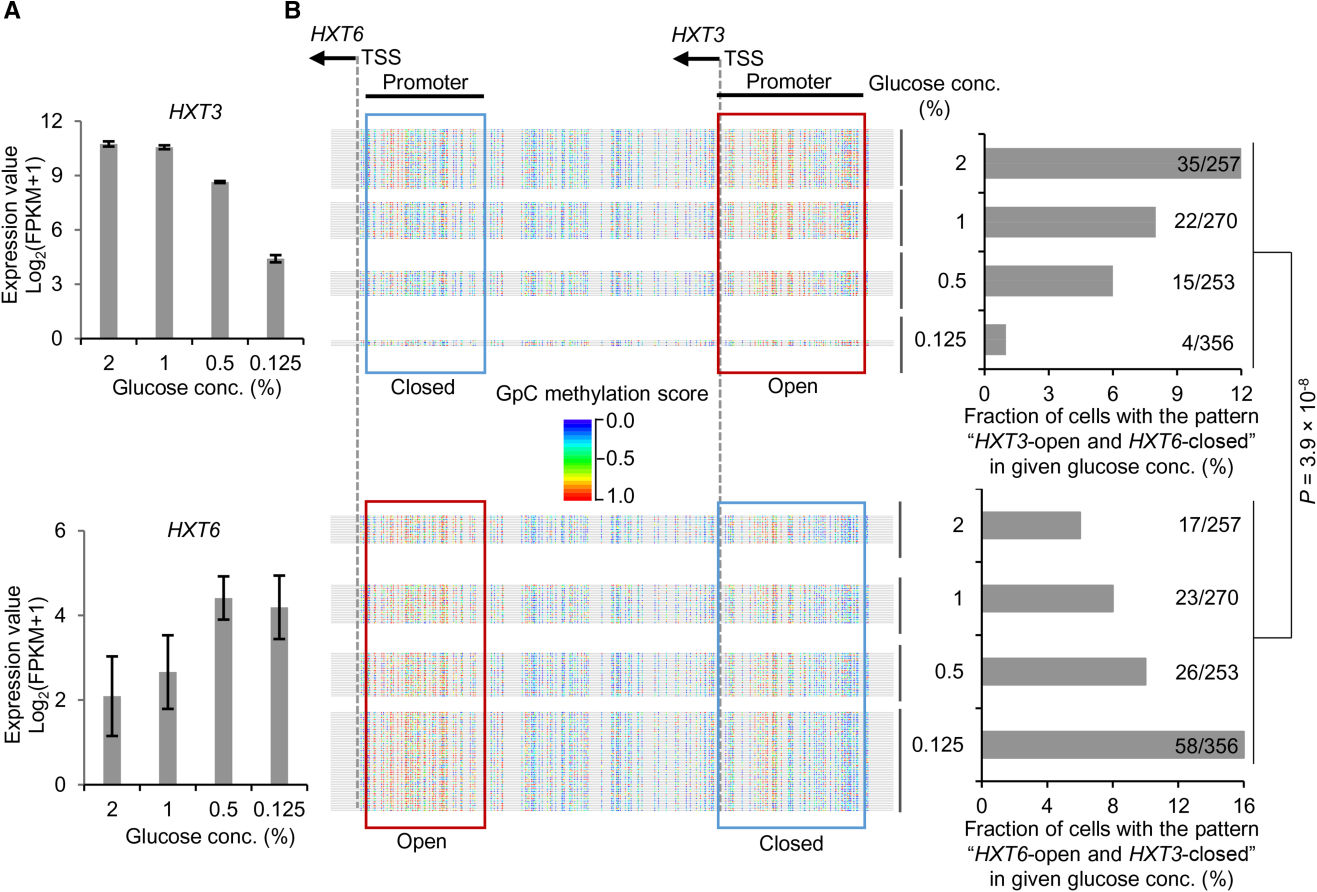
Relationship between chromatin accessibility and coexpression of *HXT3* and *HXT6*. (*A*) Expression levels of *HXT3* and *HXT6* in response to glucose concentration change. FPKM from bulk-cell RNA-seq data was taken as the expression level. (*B*) Change of the coupled chromatin statuses of *HXT3* and *HXT6* in response to different glucose concentrations. Chromatin accessibility in promoters of *HXT3* and *HXT6* at the single-cell level is shown. Each line represents a molecule (i.e., cell). GpC site is labeled as a rainbow-color dot, with methylation score from 0 (blue) to 1.0 (red). A promoter was defined as “open” (highlighted by red box) if the methylation scores of the including GpC sites had a median value greater than 0.5, and “closed” (highlighted by blue box) otherwise. Cells are shown in four groups that corresponded to four glucose concentrations. The cell fractions are also shown on the bar charts. The *P*-value was calculated by a χ^2^ test under the null hypothesis that alternative openness status of the two genes was independent of glucose concentration.

Making full use of the single-molecule and long-range advantages of MeSMLR-seq data, we explored the coupled chromatin status changes of two adjacent glucose transporter genes, *HXT3* and *HXT6*, during transcription reprogramming. The transport of glucose across the plasma membrane is the first step of glucose metabolism, and the glucose (also called hexose) transporter genes play essential regulatory roles in glucose sensing, signaling, and utilization in a yeast cell (Ozcan and Johnston 1999). Hxt3 and Hxt6 have different affinities to glucose (low-affinity for Hxt3 and high-affinity for Hxt6) and thus respond differently to the change of glucose concentration. With the decrease of glucose concentration, the expression of *HXT3* decreased, whereas *HXT6* increased, which corresponded to their low- and high-affinity to glucose (Fig. 8A).

For each glucose concentration (2%, 1%, 0.5%, and 0.125%), we counted MeSMLR-seq molecules to estimate the fractions of cell subpopulations with two opposite coupled chromatin accessibility patterns: “Open-*HXT3* and Closed-*HXT6*” and “Closed-*HXT3* and Open-*HXT6*”. The fraction of cell subpopulation with the coupled pattern “Open-*HXT3* and Closed-*HXT6*” decreased along with the reduction of glucose concentration, whereas “Closed-*HXT3* and Open-*HXT6*” increased (Fig. 8B). The changes of two coupled patterns significantly matched the expression dynamics of two genes in response to a glucose concentration change (*P*-value <3.9 × 10^−8^, χ^2^ test) (Fig. 8). These proof-of-concept results highlight the promising utility of MeSMLR-seq for studying complex epigenetic changes during transcription reprogramming.

## Discussion

In this study, we showed the consistent bulk-level nucleosome occupancy and chromatin accessibility profiles generated by MeSMLR-seq with existing methods. With the unique output of MeSMLR-seq, we investigated the organization principles of nucleosomes surrounding TSSs and studied the heterogeneity of combinatorial chromatin statuses over multiple genomic regions. Together with single-cell RNA-seq data, the relationship between chromatin accessibility and gene transcription was investigated quantitatively. Finally, we revealed the coupled chromatin changes of adjacent genes during transcription reprogramming.

A large number of studies have demonstrated key regulatory roles for nucleosome positioning and chromatin accessibility in eukaryotic gene expression (Li et al. 2005, 2007; Petesch and Lis 2008; Jiang and Pugh 2009) as well as DNA repair, recombination and other DNA-dependent processes (Lipford and Bell 2001; Dalal et al. 2007; Schwartz et al. 2009; Tilgner et al. 2009; Cole et al. 2011; Lai and Pugh 2017). The relationship between nucleosome positioning, chromatin accessibility, and gene expression has been studied most extensively (Rando and Winston 2012). However, unlike the well-studied heterogeneity of gene expression based on single-cell analyses, the heterogeneity of nucleosome positioning and chromatin accessibility is poorly studied due to limitations in experimental and sequencing techniques. Previous bulk-cell studies based on well-developed experimental techniques established the fundamental knowledge base, while their corresponding versions at the single-cell platforms have not yet led to more details. This is largely due to the sparse sequencing coverage and short read length. MeSMLR-seq provides an alternative way to address this bottleneck: Long read length guarantees the full length of the genomic region of interest (e.g., whole gene body together with the flanking neighborhood) can be covered by many single reads (that is, single DNA molecules). In the application to haploid organisms, a MeSMLR-seq read population represents the cell population, so the heterogeneity at the cell level can be investigated. In this study, MeSMLR-seq provides a long-range chromatin status landscape and nucleosome occupancy detection at the single-molecule/-cell level. The investigation of coupled chromatin changes and differential nucleosome organization principles in response to nutrition changes underline the unique MeSMLR-seq output for exploring these complex epigenetic events.

However, it should be noted that the molecule-cell link does not hold in diploid or polyploid organisms, as the molecule populations are a mix of allele-specific and cell-specific events. It leads to challenges and opportunities in the further development of new experimental (e.g., single-cell barcoding) and statistical (e.g., data deconvolution) approaches. Once cell subpopulations can be reconstructed from a molecule population, we could distinguish the allele-specific epigenome precisely from different cell subpopulations and achieve more accurate investigation of how epigenetics events behave differently at different alleles. Regardless of the wide interest in the cell-level study, the characterization of nucleosome positioning and chromatin status at single DNA molecules by MeSMLR-seq will also bring very unique and informative data to reveal the dynamic nucleosome positioning mechanism, such as assembly, disassembly, and sliding.

Besides the single-molecule information, the long length of MeSMLR-seq reads, which allows correlation analysis of exogenous and endogenous methylation statuses over different positions, could be informative for some research topics: (1) Correlation of exogenous 5mC events has shown the nucleosome occupancy pattern in this study (Fig. 2B), and thus DNA loops or other larger spatial chromatin domains that affect exogenous methylation could also be identified, which would require specific library preparation to generate even longer ONT reads; (2) as endogenous 5mC can also be detected, MeSMLR-seq can be applied to higher organisms (e.g., human) to study how methylation status at different genomic regions coordinates, but it could also provide direct evidence to address the controversial topics about how methylation status and nucleosome positioning and chromatin openness correlates. For example, in human cells, the endogenous 5mC mainly occurs at CpG sites and can be distinguished from exogenous GpC-specific 5mC detection using ONT data. Thus, the DNA methyome, nucleosome occupancy, and chromatin accessibility can be simultaneously measured on a single DNA molecule in human. Further, the ultralong length of ONT reads (up to Mbps) enables the analysis of the coupled dynamics of DNA methylation and chromatin status of adjacent genes, since the median distance between adjacent genes is 36 kb in human.

From a technical viewpoint, there are relatively few applications of ONT data in epigenetics research, as the corresponding experimental approaches or bioinformatics methods are rarely developed, although numerous applications of ONT data have been published rapidly with improved data quality and cost efficiency. In addition to the previously reported studies of identifying methylation and three-dimensional spatial organization of chromatin (https://nanoporetech.com/resource-centre/pore-c-using-nanopore-reads-delineate-long-range-interactions-between-genomic-loci), MeSMLR-seq contributes a new technique in the toolkit of single-molecule long-read sequencing to obtain first-hand details of epigenetics at single DNA molecules. More innovative studies with single-molecule long-read sequencing should be explored and expected to advance our studies to discover novel and complex biological insights.

## Methods

### Yeast strain and growth

The *Saccharomyces cerevisiae* BY4741 strain was used in this study. Yeast cells were separately grown at 30°C in media including 1% yeast extract, 2% peptone, and different carbon sources. Yeast cells were collected in the mid-log phase (OD_600_ of 0.3–0.6) and subjected to MeSMLR-seq, bulk-cell RNA-seq, and single-cell RNA-seq experiments (Supplemental Table S4; Supplemental Methods).

### MeSMLR-seq experiment

Preparation and methylation of yeast spheroplasts were performed as previously described (Fig. 1; Small et al. 2014). Briefly, yeast cells were treated with Zymolyase (final conc. = 0.25 mg/mL; amsbio) in 1 M sorbitol and 50 mM Tris (pH 7.4), and 10 mM β-mercaptoethanol. Spheroplasts were washed twice using 1 M sorbitol before methyltransferase treatment. GpC-specific methyltransferase M.CviPI (NEB) supplemented with 160 µM SAM S-adenosylmethionine was used to methylate spheroplasts at 37°C for 45 min. Genomic DNA was extracted using PCI (phenol:chloroform:isoamyl alcohol, 25:24:1) and purified by a Genomic DNA Clean & Concentrator-10 kit (Zymo Research).

We denote the above mentioned genomic DNA that undergoes in vivo spheroplast methylation as the target sample of MeSMLR-seq. Native genomic DNA extracted from yeast without M.CviPI treatment was used as the negative control (all cytosines at GpC sites are unmethylated). There is no endogenous 5mC in the yeast genome, as reported in a previous study (Capuano et al. 2014). Genomic DNA treated with M.CviPI (without spheroplast methylation) was used as a positive control (all cytosines at GpC sites are 5mCs).

The efficiency of M.CviPI methylation was evaluated using bisulfite sequencing as previously described (Small et al. 2014). First, bisulfite conversion was performed using a EZ DNA Methylation-Lightning kit (Zymo Research). Second, PCR amplification targeted to specific genomic regions was performed by ZymoTaq PreMix (Zymo Research). The *CHA1* gene region (Chr III: 15,713–16,074), *CYS3* gene region (Chr I: 130,966–131,117), *GAL10* gene region (Chr II: 278,464–278,738), and *PHO5* gene region (Chr II: 430,248–430,388) were amplified for evaluating the methylation efficiency of the positive control. The *PHO5* gene region (Chr II: 430,843–431,498), which was shown in Figure 1 of the previous study (Small et al. 2014), was used to estimate the efficiency of spheroplast methylation (i.e., the target sample of MeSMLR-seq). Third, TA cloning was performed by a TOPO TA Cloning kit (Life Technologies). Single colonies were picked and plasmids were extracted using a QIAprep Spin Miniprep kit (QIAGEN). Finally, plasmids were sequenced by Sanger sequencing. For a positive control, we estimated the efficiency of methylation as the percentage of 5mC over all GpC sites (totally 53 GpC sites for four target gene regions). Three single colonies were sequenced per gene region; and the methylation efficiency of the positive control was ([53 × 3] − 1)/(53 × 3) = 99.37%. For the target sample of MeSMLR-seq, we considered it as successfully methylated if the single colony included at least one 5mC. In total, 10 colonies were sequenced, and the methylation success rate of the target sample was up to 100% (10/10). The Sanger sequences for templates and colonies are provided in Supplemental Table S5.

Native genomic DNA (negative control), methylated genomic DNA (positive control), and extracted genomic DNA after spheroplast methylation (target sample) were directly submitted to ONT sequencing. In brief, the genomic DNA was fragmented (size = 8 kb) using Megaruptor. A sequencing library was prepared using the 1D Ligation Sequencing kit (SQK-LSK108). ONT sequencing was performed using the GridION platform with R9.4.1 flow cells.

### MeSMLR-seq data preprocessing

The software Albacore developed by ONT was used to perform base-calling for ONT raw signals. The base-called ONT sequencing data were aligned to the sacCer3 reference genome using BWA software (version 0.7.17-r1188) (Li and Durbin 2010) with the “mem” mode and the “-x ont2d” parameter. Nanopolish (version 0.8.5) (Simpson et al. 2017) with the “eventalign” mode and the “--scale-events” parameter was used to generate the alignments between event levels and 6-mers for each sequencing molecule, which were utilized for the following GpC-specific 5mC detection.

Since we used the ONT 1D sequencing strategy in this study, a DNA molecule from a yeast cell might be sequenced twice (i.e., forward and reserve strands). Thus, to achieve the “one-to-one” link between the ONT sequencing molecule and haploid yeast cell, we classified all molecules into two groups based on their aligned genomic strands: forward and reverse.

The information of MeSMLR-seq data (including data size, read length, error rate, alignment rate, and genome coverage) was summarized in Supplemental Table S1.

### GpC-specific 5mC detection at the single-molecule level and single-base resolution by MeSMLR-seq

For each 6-mer pattern that includes cytosine of GpC dinucleotide, represented here by *k*, we trained two models for the event level; one was for unmethylated cytosine and the other for methylated cytosine. We modeled the event level for unmethylated cytosine by a Gaussian distribution $N(\mu_{0},\sigma_{0}^{2})$. From the negative control data preprocessed by “nanopolish eventalign,” we pooled the event levels that were aligned to *k* and estimated *μ*_0_ and $\sigma_{0}^{2}$ by the sample mean and variance, respectively. Considering the fact that the efficiency of exogenous methylation was not always 100% (99.37% in our experiment) (Supplemental Fig. S1A, right panel), we modeled the event level for methylated cytosine by a Gaussian mixture model $\rho N(\mu_{1},\sigma_{1}^{2})+(1-\rho )N(\mu_{2},\sigma_{2}^{2})$, 0 < *ρ* < 1. The parameters *μ*_1_, *μ*_2_, $\sigma_{1}^{2}$, $\sigma_{2}^{2}$, and *ρ* were estimated based on the event levels from positive control data by EM algorithm. Denote the probability density functions of the two models as *f*_*N*_(*x*;*k*) (unmethylated cytosine) and *f*_*P*_ (*x*;*k*) (methylated cytosine), respectively. Namely,

$$f_{N}(x;k)=\frac{1}{\sqrt{2\pi }\sigma_{0}}e^{-\frac{{\left(x-\mu_{0}\right)}^{2}}{2\sigma_{0}^{2}}},$$

$$f_{P}(x;k)=\rho \frac{1}{\sqrt{2\pi }\sigma_{1}}e^{-\frac{{\left(x-\mu_{1}\right)}^{2}}{2\sigma_{1}^{2}}}+(1-\rho )\frac{1}{\sqrt{2\pi }\sigma_{2}}e^{-\frac{{\left(x-\mu_{2}\right)}^{2}}{2\sigma_{2}^{2}}}.$$

The *x* in the above formulas represents the value of the event level. The area of the overlapped region under the two probability density functions *f*_*N*_ (*x*;*k*) and *f*_*P*_ (*x*;*k*) is calculated. The discrimination of the 6-mer *k* is defined as 1 − the area of overlap.

Given a sequencing molecule from the target sample, we detected 5mC for all GpC sites. For each of the GpC sites, we listed all the 6-mers on the reference genome that cover the cytosine at the dinucleotide (Supplemental Fig. S1A, left panel). The 6-mer with >1 GpC site included or >10 aligned event levels from the molecule was excluded for 5mC detection. Among the remaining 6-mers, the one with the maximal discrimination was chosen for the calculation of methylation score. Denote *k*′ as the selected 6-mer, and *x*′ as the event level that was aligned to *k*′. The event level *x*′ was filtered out if one of log*f*_*P*_ (*x*′;*k*′) or log*f*_*N*_ (*x*′;*k*′) was <−10; otherwise, the methylation score of the GpC site was calculated as

$$s=\frac{\;f_{P}(x\prime ;k\prime )}{\;f_{P}(x\prime ;k\prime )+f_{N}(x\prime ;k\prime )}.$$

The score *s* was essentially the posterior probability of methylation given a noninformative prior. If multiple event levels were aligned to *k*′, then *f*_*P*_ (*x*′;*k*′) and *f*_*P*_ (*x*′;*k*′) were replaced by the product of the multiple likelihood.

As a cross-validation, we randomly split each of the negative control and positive control data into two halves. One of the halves was used to train the models. Using the trained models, we detected 5mC on the other half. With the real methylation status of the test data being known, we were able to evaluate the detection results. It turned out that the area under the ROC curve was 0.86 (Fig. 2A).

### Nucleosome occupancy detection at the single-molecule level by MeSMLR-seq

We developed a bioinformatics method, named NP-SMLR, to detect and phase nucleosomes at the single-molecule level (Fig. 2C; Supplemental Code).

Let *X*_1_ *X*_2_…*X*_*l*_ be a molecule, where *X*_*i*_ is the *i*-th base. Denote *s*_*i*_ as the methylation score of *X*_*i*_, if *X*_*i*_ is the cytosine of the GpC dinucleotide. Suppose that the event levels of all GpC sites are independent. Nucleosome occupancy detection refers to finding a path ****π**** = *π*_1_*π*_2_…*π*_*l*_ that maximizes the likelihood of signals

$$\pi^{*}=\underset{\pi }{\mathrm{argmax}}\overset{n}{\underset{t=1}{\prod }}\mathrm{Pr}(s_{i_{t}}|\pi_{i_{t}}).$$

*π*_*i*_ takes the value from {*L*, *N*_1_, *N*_2_, …, *N*_147_}. *L* represents the linker region; *N*_*m*_ represents the *m*-th base within a nucleosome; *i*_1_, *i*_2_, …, *i*_*n*_ are the positions of cytosines that belong to GpC dinucleotides. The elements of path ****π**** are restricted such that: (1) *N*_*m*_ is followed by *N*_*m*+1_ (1 ≤ *m* ≤ 146); (2) *N*_147_ is followed by *L*; and (3) *L* is followed by *L* or *N*_1_.

Based on the methylation scores of all GpC sites from all molecules in negative and positive control training data, we can fit two density curves using the “density” command in R (version 3.3.0; R Core Team 2016), respectively. The two density functions are denoted as *q*_*N*_ (·) and *q*_*P*_ (·), respectively (Supplemental Fig. S1B). A dummy methylation score *s*_*i*_ = −1 is added for *X*_*i*_ if it is not a cytosine of GpC dinucleotide. Define

$$p_{i}(\pi_{i})≜1_{\left\{s_{i}=-1\right\}}+1_{\left\{s_{i}\neq -1\right\}}\cdot \mathrm{Pr}(s_{i_{t}}|\pi_{i_{t}})=1_{\left\{s_{i}=-1\right\}}+1_{\left\{s_{i}\neq -1\right\}}\cdot q_{P}{\left(s_{i}\right)}^{1_{\left\{\pi_{i}=L\right\}}}\cdot q_{N}{\left(s_{i}\right)}^{1_{\left\{\pi_{i}\neq L\right\}}}.$$

Let $a_{\pi_{i},\pi_{i+1}}$ be the compatibility indicator of two adjacent states such that

$$a_{\pi_{i},\pi_{i+1}}=1_{\left\{\pi_{i}=N_{m},\pi_{i+1}=N_{m+1},1\leq m\leq 146\right\}}+1_{\left\{\pi_{i}=L,\pi_{i+1}=N_{1}\right\}}+1_{\left\{\pi_{i}=L,\pi_{i+1}=L\right\}}.$$

The objection function can therefore be expressed as

$$L=p_{1}(\pi_{1})\overset{n}{\underset{i=2}{\prod }}p_{i}(\pi_{i})a_{\pi_{i-1},\pi_{i}}.$$

Define

$$\ell_{k,\zeta }=\underset{\pi_{1}\cdots \pi_{k},\pi_{k}=\zeta }{\max}p_{1}(\pi_{1})\overset{k}{\underset{i=2}{\prod }}p_{i}(\pi_{i})a_{\pi_{i-1},\pi_{i}}.$$

Then, the maximum of objection function can be obtained by iteration

$$\ell_{k+1,\xi }=\underset{\zeta }{\max}\ell_{k,\zeta }\cdot p_{k+1}(\xi )\cdot a_{\zeta ,\xi },$$

$$\underset{\pi }{\max}L=\underset{\xi }{\max}\ell_{n,\xi }.$$

Accordingly, π* can be obtained through dynamic programming (Fig. 2C). We start by building an *l* × 148 matrix *V*. Line *i* corresponds to *X*_*i*_, the *i*-th base of the molecule. Column 1 corresponds to the linker, and the other columns (from Column 2 to Column 148) correspond to *N*_1_, *N*_2_, …, *N*_147_, separately. Initialize *V*[1, 1] = *p*_1_(*L*), and *V*[1, *j*] = *p*_1_(*N*_*j*−1_), 2 ≤ *j* ≤ 148. Elements in Line *i*(2 ≤ *i* ≤ *n*) are then calculated iteratively. For Column 1, the element *V*[*i*, 1] is set as max{*V*[*i* − 1, 1], *V*[*i* − 1, 147]}*q*_*P*_(*s*_*i*_) if *X*_*i*_ is cytosine of GpC, or max{*V*[*i* − 1, 1], *V*[*i* − 1, 147]} otherwise. For Column *j* (2 ≤ *j* ≤ 148), *V*[*i*, *j*] is set as *V*[*i* − 1, *j* − 1]*q*_*N*_(*s*_*i*_), or *V*[*i* − 1, *j* − 1] otherwise. When updating an element, we record the position of the previous element that leads to the maximal value and store the position as a pointer. After updating all elements, the maximal element in the last line is found (elements that equal to 1 are not considered), and the nucleosome occupancy detection is completed through the backtracking of pointers. All calculations are performed in log scale to avoid rounding error.

We evaluated the accuracy of nucleosome occupancy detection (NP-SMLR) through simulation tests under different nucleosome coverage and GpC frequency (Fig. 2D). In detail, DNA sequence (3-kb length) was simulated with randomly assigned GpC sites at a given frequency. Lengths of linkers between nucleosomes were sampled independently and sequentially. Each time, the linker length was sampled from the normal distribution $N(\nu_{1},\gamma_{1}^{2})$ with probability *τ*, and $N(\nu_{2},\gamma_{2}^{2})$ with probability 1 − *τ*, corresponding to regular nucleosome array and open region with specific biological functions, respectively. We set *ν*_2_ > *ν*_1_ and $\gamma_{2}^{2}>\gamma_{1}^{2}$. Nucleosomes were then placed on the DNA sequence, with their distance being set as the above simulated linker length. Methylation scores for GpC sites occupied by nucleosomes were generated based on the score distribution of negative control data, whose density function was *q*_*N*_ (·). For GpC sites within linkers, *q*_*P*_ (·) was used instead. NP-SMLR was applied on the simulated sequence. Denote $\hat{Z_{i}}$ and *Z*_*i*_ as the predicted and real indicators of whether the *i*-th base locates in nucleosome or not, respectively. The accuracy was defined as

$$A=\frac{1}{l}\overset{l}{\underset{i=1}{\sum }}1_{\left\{\hat{Z_{i}}=Z_{i}\right\}},$$

where *l* is the length of the simulated DNA sequence. In simulation tests, we set *ν*_1_ = 15, *γ*_1_ = 5, *γ*_2_ = 10, *τ* = 0.1. We set *ν*_2_ as 15, 50, 100, 200, 300, 400, 500, and 600, respectively, to achieve different nucleosome coverage (defined as the proportion of bases covered by nucleosomes). For each parameter setting, the above simulation was carried out 1000 times.

### Bulk-cell level nucleosome occupancy analyses based on MeSMLR-seq data

The genomic coordinates of all nucleosomes predicted by NP-SMLR at the single-molecule level were pooled and subjected to iNPS software (version 1.2.2) (Chen et al. 2014) with default parameters to generate bulk-cell level nucleosome occupancy profiles and to call nucleosome peaks.

The nucleosome occupancy profiles were used to generate Figure 3, A and B, Figure 4, D and E (upper panel), Supplemental Figure S5, A and B, and Supplemental Figure S6, D and E. The nucleosome peaks called by iNPS were used for the comparison with MNase-seq (Fig. 3C).

### Measurement of nucleosome positioning heterogeneity

The heterogeneity of nucleosome positioning was measured by the variation of the +1 nucleosome positioning relative to the TSS across different cells (Fig. 4B; Supplemental Fig. S2A). For each molecule/cell, we first defined the nucleosome whose center was located downstream from the TSS and closest to the TSS as the +1 nucleosome. Next, we sorted the distances between the +1 nucleosome and the TSS and removed the upper 10% values for robustness. The standard variance of the remaining values was used to represent the heterogeneity of nucleosome positioning for each gene.

### Measurement of nucleosome spacing uniformity

The uniformity of nucleosome spacing was measured by the variation of the distance between adjacent nucleosomes (i.e., the length of linker region) (Fig. 4C; Supplemental Fig. S2B). For each gene, the molecules that fully covered the region (from 500 bp upstream of to 100 bp downstream from the TSS) were chosen. For each molecule, we calculated the lengths of all linker regions that were located in the region “−500, +100”. Then, we calculated the absolute deviation of linker length pair-wisely. The sum of the deviation values was divided by the number of linker pairs. The obtained value, which described the variation of nucleosome distance, was namely the nucleosome spacing uniformity.

### Chromatin accessibility mapping at the single-molecule level based on MeSMLR-seq data

Based on the methylation scores of all GpC sites per molecule, we detected accessible chromatin regions along the molecule. Given a single molecule *X*_1_ *X*_2_…*X*_*l*_, where *X*_*i*_ is the *i*-th base, we defined the interval from *X*_*i*_ to *X*_*j*_ as an accessible region if: (1) *X*_*i*_ and *X*_*j*_ were adjacent GpC sites; (2) the corresponding methylation scores *s*_*i*_ and *s*_*j*_ were >0.5; and (3) the distance between *X*_*i*_ and *X*_*j*_ was <100 bp. The continuous accessible regions were merged. Given an accessible region, the chromatin accessibility score was defined as the median methylation score among all GpC sites within this region.

In this study, we only considered the accessible regions with the length ≥100 bp for each molecule. A genome-wide chromatin accessibility profile was generated through merging accessible regions of all molecules. The chromatin accessibility profile was used to generate Figure 5, A and B, Figure 6 (upper panel), Supplemental Figure S4, A and B, Supplemental Figure S5C, Supplemental Figure S6, B and C, and Supplemental Figure S7A.

### Chromatin accessibility peak calling at the bulk-molecule/-cell level based on MeSMLR-seq data

We defined significantly accessible genomic regions as described in the previous study (Grünberg et al. 2016). Let *G*_*i*_ be the *i*-th base of the genome. Denote $X_{i}^{\left(1\right)},X_{i}^{\left(2\right)},\ldots ,X_{i}^{\left(M\right)}$ as the bases from *M* sequencing molecules that covered *G*_*i*_, and $s_{i}^{\left(1\right)},s_{i}^{\left(2\right)},\ldots ,s_{i}^{\left(M\right)}$ as the corresponding methylation scores if *G*_*i*_ is a GpC site. Define $r_{i}=1/M\sum_{\;j=1}^{M}1_{\left\{s_{i}^{\left(j\right)}>0.5\right\}}$, which is the ratio of methylated bases (methylation score >0.5), and denote $\bar{r}$ as the average of ratios of all GpC sites. We defined the interval between *G*_*i*_ and *G*_*j*_ as a significantly accessible region if: (1) *G*_*i*_ and *G*_*j*_ were adjacent GpC sites; (2) $r_{i}>1.5\bar{r}$, and $r_{j}>1.5\bar{r}$; and (3) the distance between *G*_*i*_ to *G*_*j*_ was <100 bp. The continuous accessible regions were merged to generate a longer accessible genomic region (referred to as “chromatin accessibility peak”).

In this study, we only considered the peaks with the length ≥100 bp. For sequencing molecules aligned to forward and reverse genomic strands, we defined chromatin accessibility peaks separately. The overlapped peaks between the forward and reverse strands were used for the comparison with two existing methods (i.e., ATAC-seq and DNase-seq) (Fig. 5C).

### Definition of gene promoter region and measurement of gene accessibility

To quantitatively measure the accessibility of genes, we first defined the promoter region for each gene. Briefly, chromatin accessibility peaks (including both forward and reverse strands) were called using MeSMLR-seq data for each biological sample. For each biological sample, the overlapped peaks between forward and reverse strands for MeSMLR-seq were merged together. Next, we combined the merged peaks of MeSMLR-seq from all biological samples and the overlapped peaks between two biological replicates of DNase-seq. For each gene, (1) if there was only one peak that was located within the upstream 500 bp and downstream 100 bp of the TSS (named “−500, +100” region), the peak was defined as the promoter region; or (2) if there were multiple peaks that were located in the “−500, +100” region, the peak that had the longest overlap was defined as the promoter region; or (3) if there was no peak locating in the region “−500, +100”, the region “−500, +100” was defined as the promoter region.

At the single-molecule level, the accessibility score of a gene was calculated as the median methylation score among all GpC sites within the promoter region. For all molecules covering the promoter of a given gene, we categorized them into two chromatin statuses: “open” if the accessibility score was >0.5; “closed” otherwise. The defined promoter region and the corresponding accessibility score were used to generate Figure 5E, Figure 6 (upper panel), Figure 7, Figure 8, Supplemental Figure S6A, and Supplemental Figure S7D.

### Analyses of dynamic gene expression and chromatin accessibility among three carbon sources

Differentially expressed genes were identified using Cuffdiff (version 2.2.1) (*q*-value <0.01) (Trapnell et al. 2010) between glucose (Glu) and other two carbon sources, galactose (Gal) and raffinose (Raf). Overall, there were 700 up-regulated and 682 down-regulated genes in Gal (Glu vs. Gal) and 605 up-regulated and 727 down-regulated genes in Raf (Glu *vs.* Raf). These differentially expressed genes were used to generate Supplemental Figure S7, B–D. Gene enrichment analyses in Supplemental Figure S7C were performed using DAVID (version 6.8) (Huang da et al. 2009).

For the differential chromatin accessibility analyses, we first calculated the bulk-cell-level chromatin accessibility as the ratio of those with “open” status among the molecules that fully covered the gene promoter. For each gene, the differential chromatin accessibility score was computed as the difference of bulk-cell-level chromatin accessibility between two carbon sources (Glu minus Gal for “Glu vs. Gal”; Glu minus Raf for “Glu vs. Raf”).

## Data access

The MeSMLR-seq data generated in this study have been submitted to the NCBI BioProject database (https://www.ncbi.nlm.nih.gov/bioproject/) under accession number PRJNA510813. The bulk-cell RNA-seq and the single-cell RNA-seq data generated in this study have been submitted to the NCBI Gene Expression Omnibus (GEO; https://www.ncbi.nlm.nih.gov/geo/) under accession number GSE131702. The Sanger sequences data generated in this study are summarized in Supplemental Table S5. The public sequencing data used in this study are summarized in Supplemental Table S6. The source code of NP-SMLR is in the Supplemental Material (Supplemental_Code.zip) and is also available at https://github.com/Au-Lab/NP-SMLR.

## Supplementary Material

Supplemental Material
