# Supplementary material for: Single-molecule long-read sequencing reveals the chromatin basis of gene expression
Source: Genome Res. 2019 Aug;29(8):1329–42. doi: 10.1101/gr.251116.119 (PMC6673713; doi:10.1101/gr.251116.119)
Supplement: Supplemental Material [file supp_29_8_1329__index.html]

Single-molecule long-read sequencing reveals the chromatin basis of gene expression — Supplemental Material 

# Single-molecule long-read sequencing reveals the chromatin basis of gene expression

## Supplemental Material

- Supplemental\_Materials.pdf
- Supplemental\_Code.zip
- Supplemental\_Table\_S5.xlsx
