# Supplementary material for: Single-molecule long-read sequencing reveals the chromatin basis of gene expression
Source: Genome Res. 2019 Aug;29(8):1329–42. doi: 10.1101/gr.251116.119 (PMC6673713; doi:10.1101/gr.251116.119)
Supplement: Supplemental Material [file supp_gr.251116.119_Supplemental_Materials.pdf]

Supplemental Materials

for

Single-molecule long-read sequencing reveals the chromatin basis of gene expression

Yunhao Wang[a,b,1], Anqi Wang[a,b,1], Zujun Liu[a,b], Andrew L. Thurman[b], Linda S. Powers[b], Meng Zou[b], Yue Zhao[a,b], Adam Hefel[b], Yunyi Li[b], Joseph Zabner[b], Kin Fai Au[a,b,c,2]

[a] Department of Biomedical Informatics, The Ohio State University, OH 43210, USA

[b] Department of Internal Medicine, University of Iowa, Iowa City, IA 52242, USA

[c] Department of Biostatistics, University of Iowa, Iowa City, IA 52242, USA

[1] Y. W. and A. W. contributed equally to this work.

[2] To whom correspondence should be addressed. E-mail: kinfai.au@osumc.edu

Contents

1. Supplemental Notes

2. Supplemental Methods

3. Supplemental Figures S1-S7

4. Supplemental Tables S1-S4, S6, S7

Supplemental Notes

Note 1: Promoter openness and gene transcription

Using the MeSMLR-seq data, we generated the nucleosome occupancy profiles surrounding the TSSs of all protein-coding genes. Consistent with previous studies (Yuan et al. 2005; Hughes and Rando 2014), MeSMLR-seq data showed that highly-expressed genes had more pronounced nucleosome-depletion region in the upstream of TSS and well-positioned nucleosome array across gene body (Supplemental Fig. S5A, B). Nucleosome occupancy of the genes with high expression levels showed an obvious drop at TSS and distinct peaks within gene body, while such tendency was mild for the genes with the lower 25th percentile expression level (Supplemental Fig. S5B).

In addition to nucleosome occupancy, the chromatin accessibility profiles by MeSMLR-seq showed that the promoter regions of the highly-expressed genes were more accessible than the lowly-expressed genes (Supplemental Fig. S5C). It indicates the critical role of promoter accessibility on gene transcription regulation. We further examined the chromatin statuses of the binding regions of several important transcriptional regulators, including RNA polymerase II (Pol2), five general regulatory factors (Abf1, Cbf1, Mcm1, Rap1 and Reb1) and two mediators (Med8 and Med17) (Supplemental Methods) (Park et al. 2013; Grunberg et al. 2016; Rossi et al. 2018). The enrichment signal of Pol2 in gene body was positively correlated with chromatin accessibility of gene promoter (Supplemental Fig. S6A). The binding regions of the other regulatory factors and mediators were relatively accessible and nucleosome-evicted, which allows the assembly of transcription initiation complex (Supplemental Fig. S6B-E).

Note 2: Dynamic change of chromatin status in response to different carbon sources

We next sought to investigate the dynamics of chromatin status during transcription changes in response to different nutrition conditions. Carbon source is the basic nutrition and is essential for yeast growth (Paulo et al. 2015). In addition to glucose (Glu), which is the preferred carbon source for S. cerevisiae, we grew yeast cells separately using galactose (Gal) and raffinose (Raf) carbon sources, and generated both MeSMLR-seq and RNA-seq data. Compared to those under Gal and Raf conditions, yeast cells under Glu showed more accessible promoter (Supplemental Fig. S7A). 21.62% (1,384 of 6,713) of protein-coding genes were differentially expressed between Glu and Gal, and 20% (1,332 of 6,713) between Glu and Raf, which indicated significant transcription reprogramming in response to different carbon sources (Supplemental Fig. S7B). The up-regulated genes in Glu compared to Gal or Raf were mainly located in cytoplasm and involved in the biogenesis of ribosomes (Supplemental Fig. S7C). In contrast, the up-regulated genes in both Gal and Raf conditions compared to Glu were significantly related to the oxidation-reduction process and carbon metabolism, and were located in mitochondrion. Those significantly up-regulated genes in Glu underwent more difference of chromatin accessibility in their promoters (P-value=1.2×10^{-14} for Glu vs. Gal, P-value=3.6×10^{-11} for Glu vs. Raf, Wilcoxon rank sum test, Supplemental Fig. S7D), which contributed the overall high chromatin accessibility in the preferred carbon source (Glu) over Gal and Raf (Supplemental Fig. S7A).

Supplemental Methods

Analyses of ATAC-seq, DNase-seq, MNase-seq, ChIP-seq, ChIP-exo and ChEC-seq data

The information (including yeast strain, growth condition, GEO accession number, data format and reference) of public sequencing data used in this study was summarized in Supplemental Table S6.

Quality control of raw sequencing data (FASTQ format) was performed using FastQC and cutadapt; and alignment was performed using Bowtie2 software (version 2.2.5) (Langmead and Salzberg 2012) with default parameters.

For ATAC-seq (Schep et al. 2015) and ChIP-seq (Pol2) (Park et al. 2013) data, MACS2 software (version 2.2.1) (Zhang et al. 2008) with default parameters was used to call significantly-enriched peaks (*q*-value <0.05).

For MNase-seq data (Weiner et al. 2015), iNPS (Chen et al. 2014)with default parameters was used for nucleosome calling.

For DNase-seq data (Zhong et al. 2016), F-Seq software (version 1.85) (Boyle et al. 2008) with default parameters was used to call significantly-enriched peaks (peak length ≥100 bp).

For ChIP-exo (Abf1, Cbf1, Mcm1, Rap1 and Reb1) data, the called peak files were directly downloaded from the original study (Rossi et al. 2018).

For ChEC-seq (Med8 and Med17) data (Grunberg et al. 2016), chec-seq script (https://github.com/zentnerlab/chec-seq) was used to call significantly-enriched peaks (signal-noise ratio ≥10 and peak length ≥100 bp).

Correlation and overlapping analyses between MeSMLR-seq and MNase-seq

For correlation analysis of the bulk-cell level nucleosome occupancy results, we used iNPS to generate nucleosome occupancy profiles (BigWig format) for MNase-seq and MeSMLR-seq, respectively. Pearson correlation coefficient of nucleosome occupancy profiles (across whole genome and bin size as 10 bp) was calculated between two methods (Fig. 3A).

For overlapping analysis of nucleosomes, we only considered the two nucleosome peaks (from MeSMLR-seq and MNase-seq, respectively) as overlapped if ≥50% region of one peak was covered by another peak (Fig. 3C).

Correlation and overlapping analyses among MeSMLR-seq, ATAC-seq and DNase-seq

For correlation analysis of the bulk-cell level chromatin accessibility results, we generated genome-wide chromatin accessibility profiles (BigWig format) for three methods, separately. Pearson correlation coefficients of chromatin accessibility profiles (across the whole genome and bin size of 10 bp) were calculated among three methods (Fig. 5A).

For MeSMLR-seq data, we separately called significantly-enriched peaks for molecules aligned to forward and reverse strands. Only the overlapped peaks between the forward

and reverse strands for MeSMLR-seq data, and the overlapped peaks between two biological replicates for ATAC-seq and DNase-seq were used for overlapping analysis (Fig. 5C).

Single-cell RNA-seq experiment and data analysis

Yeast cells growing in YPD (1% yeast extract, 2% peptone and 2% glucose) medium were collected and spheroplasts were prepared as described above. Cell viability was measured using Trypan blue exclusion method and cell number was counted by hemocytometer. Of note, considering the fragility of spheroplasts, we modified the loading strategy of buffer before running the 10X ChromiumTM Controler (10X Genomics). Firstly, Single Cell Master Mix (10X Single Cell 3' Reagent Kit v2) was prepared and added into Single Cell A Chip. Next, instead of nuclease-free water, sorbitol was added (final conc. = 1 M) and mixed well. Finally, spheroplasts suspended in 1 M sorbitol were added. In total, 318 million read pairs (2 x 150 bp) were generated by Illumina HiSeq 4000 platform.

The quality of single-cell RNA-seq (scRNA-seq) data was evaluated by FastQC software. Cellranger software (version 2.1.1) with default parameters was used to process scRNA-seq data and generate the gene-cell matrix. For quality control of scRNA-seq data, we excluded the cells with >10,000 UMI (unique molecular identifier) counts as they were potentially from artificial cell or cell duplets (Stegle et al. 2015). After quality control, 2,812 single cells with 4,335 UMI counts (median value) per cell and 103,002 read pairs (median value) per cell were used in the following analyses. The number of expressed genes (≥1 UMI) per cell was 1,572 (median value). DESeq2 package (Anders and Huber 2010) was used to normalize scRNA-seq UMI count data for 2,812 cells.

Bulk-cell RNA-seq experiment and data analysis

Total RNA was extracted using Quick-RNA Fungal/Bacterial Miniprep Kit (Zymo Research). Sequencing library was prepared using TruSeq Stranded mRNA Library Prep Kit and 10 million read pairs (2 x 150 bp) on average per sample were generated using Illumina HiSeq 4000 platform. Three biological replicates per biological condition were performed.

The quality of bulk-cell RNA-seq data was evaluated by FastQC software (version 0.11.3, https://www.bioinformatics.babraham.ac.uk/projects/fastqc/) and sequencing adaptors were trimmed by Cutadapt software (version 1.8.1) (Marcel 2011). Processed reads were aligned to reference genome (version UCSC sacCer3) by Hisat2 software (version 2.0.0-beta) (Kim et al. 2015) with default parameters. Cufflinks (version 2.2.1) (Trapnell et al. 2010) with default settings were separately used for quantifying gene expression, normalizing gene expression and analyzing differential gene expression. The FPKM (Fragments Per Kilobase Million) value was calculated as the expression level of genes. The cutoff of statistical significance for differential gene expression analysis was q-value < 0.01.

The bulk-cell RNA-seq data was summarized in the Supplemental Table S7.

Supplemental Figures

Figure S1

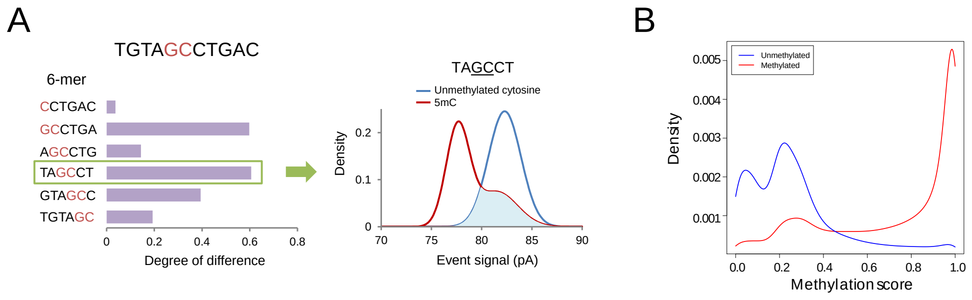

Fig. S1 5mC methylation calling at GpC sites and distribution of methylation scores. (A) An example showing the difference on event level distribution of a 6-mer with unmethylated cytosine or 5mC at GpC site (right panel). Among all 6-mers covering a GpC site, the one with the largest degree of difference was chosen for methylation detection (left panel). **(B)** The probability distribution of methylation scores for negative and positive control data. The figure was drawn based on the data that were used for 5mC detection test.

Figure S2

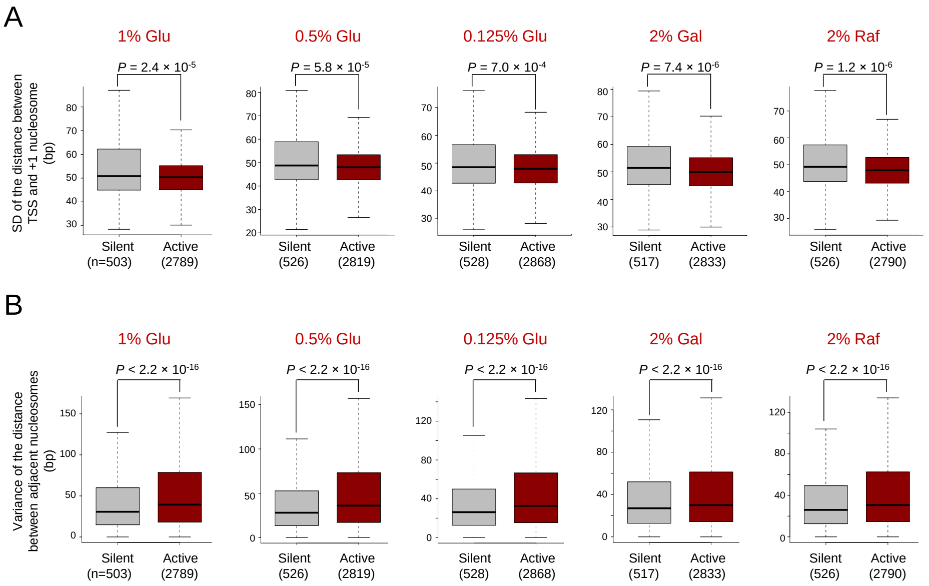

Fig. S2 Heterogeneity of nucleosome positioning and uniformity of nucleosome spacing. **(A)** Heterogeneity of nucleosome positioning for five growth conditions. The heterogeneity of nucleosome positioning was measured by the standard deviation of the distances between +1 nucleosome and TSS. SD, standard deviation. The *P*-value was calculated by Wilcoxon rank sum test. **(B)** Uniformity of nucleosome spacing for five growth conditions. The *P*-value was calculated by Wilcoxon rank sum test.

Figure S3

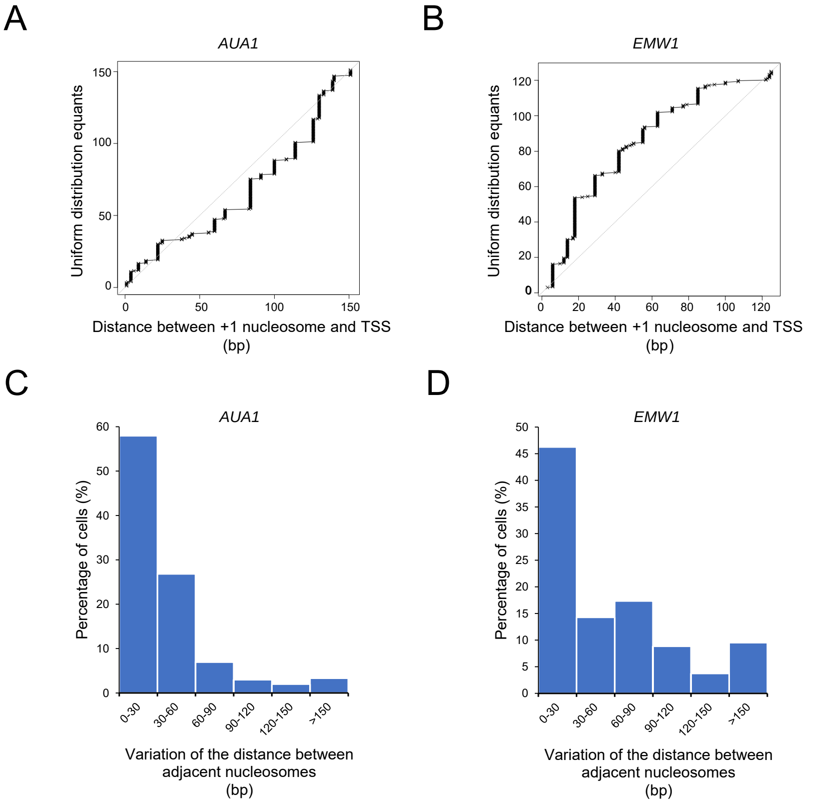

Fig. S3 Differential nucleosome organization between silent (*AUA1*) and active (*EMW1*) genes. (A, B) Q-Q plot illustration of the heterogeneity of nucleosome positioning. Each cross mark represents a molecule/cell. The *x*-axis is the distance between +1 nucleosome and TSS. The *y*-axis is the equant under the assumption that all distance values are evenly distributed. **(C, D)** Uniformity of nucleosome spacing. Smaller variation (*x*-axis) indicates that nucleosomes are more likely to be uniformly spaced.

Figure S4

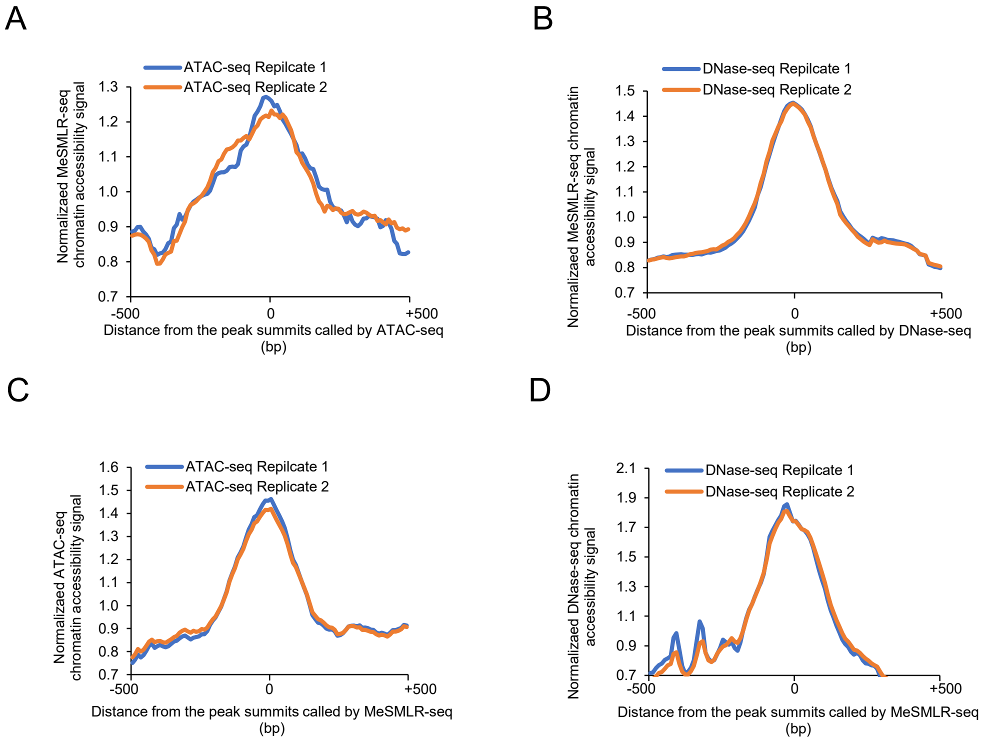

Fig. S4 Consistent profiles of chromatin accessibility among MeSMLR, ATAC-seq and DNase-seq. (A) MeSMLR-seq signal distribution surrounding the peak summits called by ATAC-seq. **(B)** MeSMLR-seq signal distribution surrounding the peak summits called by DNase-seq. **(C)** ATAC-seq signal distribution surrounding the peak centers called by MeSMLR-seq. **(D)** DNase-seq signal distribution surrounding the peak centers called by MeSMLR-seq.

Figure S5

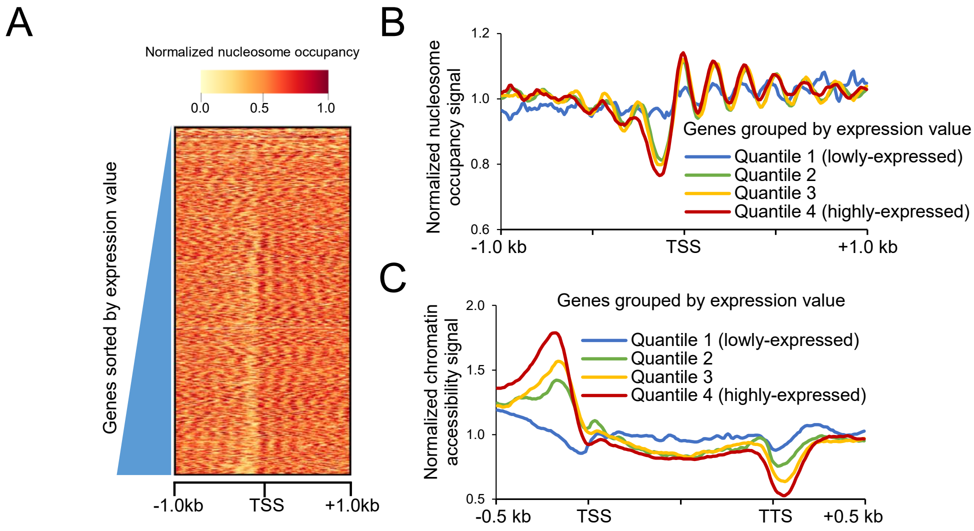

Fig. S5 Relationship between nucleosome occupancy, chromatin accessibility and gene expression. (A) Nucleosome occupancy profiles across all protein-coding genes with the ascending order of gene expression level from top to bottom. **(B)** Nucleosome occupancy profiles at the bulk-cell level for protein-coding genes with different expression levels. **(C)** Chromatin accessibility profiles at the bulk-cell level for protein-coding genes with different expression levels.

Figure S6

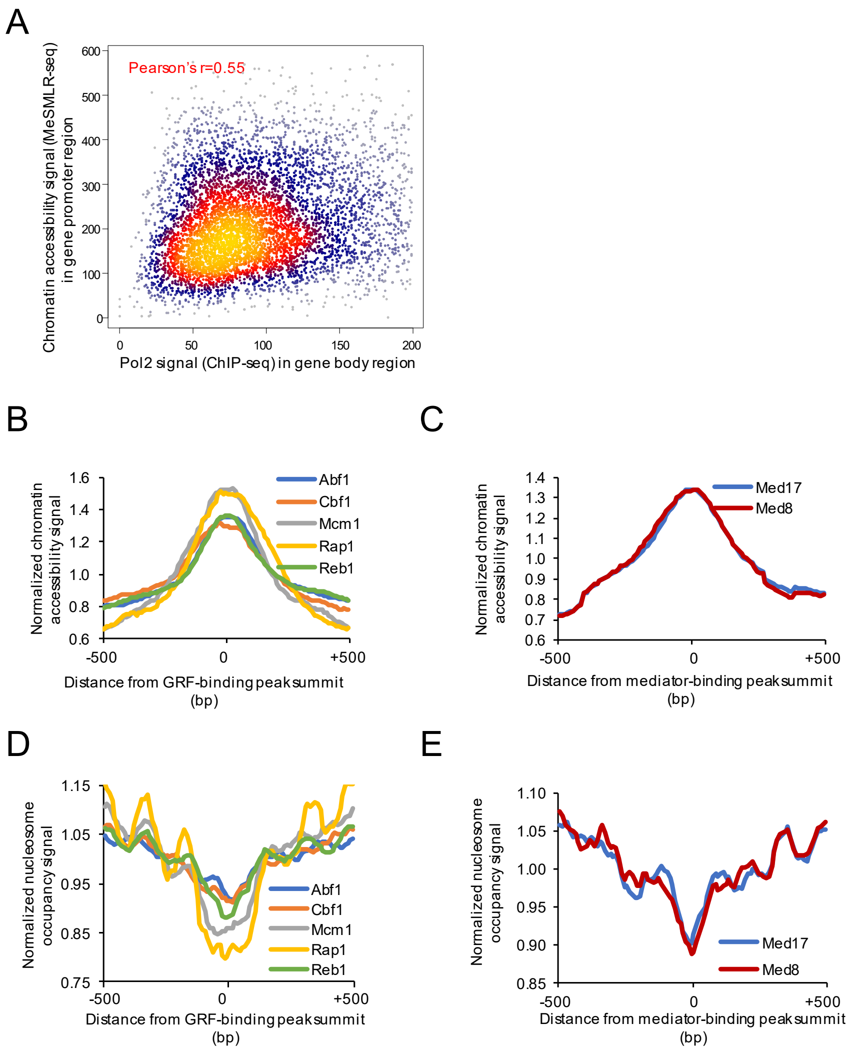

Fig. S6 Chromatin accessibility and nucleosome occupancy profiles at the binding sites of transcription-related factors. (A) Correlation between chromatin accessibility in promoter and Pol2 binding signal in gene body. Each point represents one gene. **(B, D)** Chromatin accessibility **(B)** and nucleosome occupancy **(D)** profiles at the binding sites of five general regulatory factors. **(C, E)** Chromatin accessibility **(C)** and nucleosome occupancy **(E)** profiles at the binding sites of two mediators.

Figure S7

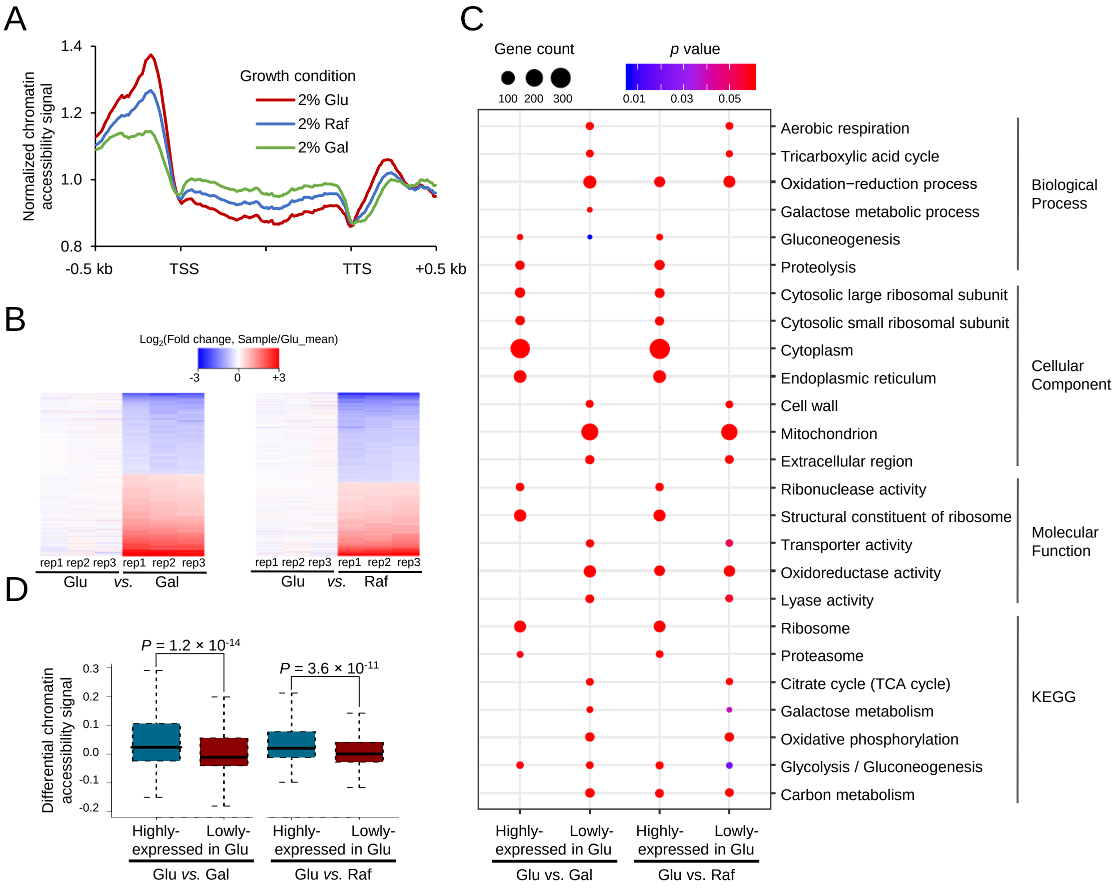

Fig. S7 Differential chromatin accessibility and gene expression under different carbon sources. **(A)** Differential chromatin accessibility patterns under glucose, galactose and raffinose. **(B)** Differential gene expression patterns under different growth conditions. Fold change = (the FPKM value of the sample)/(the averaged FPKM under glucose condition). **(C)** Gene enrichment analyses for differentially-expressed genes. **(D)** Difference of chromatin accessibility between up- and down-regulated genes under different carbon sources. The *P*-value was calculated by Wilcoxon rank sum test. Glu, glucose; Gal, galactose; and Raf, raffinose.

Supplemental Tables

Table S1. Statistics of MeSMLR-seq data generated in this study

Sample	Aligned strand	Number of aligned reads	Alignment rate of bases (%)	Error rate of reads (%)	Genome coverage (×)	Length of sequencing reads (kb)			
						Max	Median	Mean	Standard derivation
Positive control	Forward	456833	96.99	20.48	278.90	55.92	7.45	7.37	3.18
	Reverse	455786	97.01	20.44	278.18	42.88	7.44	7.37	3.17
	Forward+Reverse	912619	97.00	20.46	557.08	55.92	7.45	7.37	3.17
Negative control	Forward	619320	97.03	15.33	371.09	59.30	7.37	7.23	2.96
	Reverse	619326	97.04	15.33	371.00	46.87	7.36	7.23	2.96
	Forward+Reverse	1238646	97.04	15.33	742.09	59.30	7.37	7.23	2.96
2% Glu	Forward	711608	97.95	15.47	410.10	42.97	7.18	6.96	3.23
	Reverse	713840	97.99	15.50	411.01	38.45	7.17	6.95	3.23
	Forward+Reverse	1425448	97.97	15.49	821.11	42.97	7.18	6.95	3.23
1% Glu	Forward	640276	97.52	17.29	360.59	45.75	7.11	6.80	3.52
	Reverse	642098	97.57	17.32	361.89	35.52	7.12	6.80	3.51
	Forward+Reverse	1282374	97.54	17.30	722.48	45.75	7.12	6.80	3.52
0.5% Glu	Forward	597399	97.60	13.95	331.82	43.48	6.95	6.70	3.31
	Reverse	599093	97.70	13.99	332.73	34.26	6.95	6.70	3.32
	Forward+Reverse	1196492	97.65	13.97	664.55	43.48	6.95	6.70	3.31
0.125% Glu	Forward	734748	97.86	13.22	417.95	50.80	7.02	6.87	3.15
	Reverse	733380	97.94	13.26	417.04	52.60	7.01	6.86	3.15
	Forward+Reverse	1468128	97.90	13.24	835.00	52.60	7.02	6.87	3.15
2% Gal	Forward	527214	97.76	15.55	272.92	63.14	6.42	6.25	2.83
	Reverse	528143	97.86	15.59	273.48	59.10	6.43	6.25	2.82
	Forward+Reverse	1055357	97.81	15.57	546.40	63.14	6.43	6.25	2.83
2% Raf	Forward	697945	97.33	16.66	308.83	40.11	5.19	5.34	3.35
	Reverse	698648	97.42	16.66	309.81	36.33	5.22	5.35	3.35
	Forward+Reverse	1396593	97.37	16.66	618.64	40.11	5.20	5.35	3.35

Table S2. Number of nucleosomes phased by single molecules of MeSMLR-seq data

Sample	Aligned strand	Number of genes covered by single molecules			
		Maximal	Median	Mean	Standard derivation
2% Glu	Forward	244	37	35	18
	Reverse	226	37	36	18
	Forward+Reverse	244	37	35	18
1% Glu	Forward	271	36	34	19
	Reverse	207	36	34	19
	Forward+Reverse	271	36	34	19
0.5% Glu	Forward	256	36	34	19
	Reverse	177	36	34	19
	Forward+Reverse	256	36	34	19
0.125% Glu	Forward	294	37	36	18
	Reverse	258	37	36	18
	Forward+Reverse	294	37	36	18
2% Gal	Forward	306	32	31	16
	Reverse	356	32	31	16
	Forward+Reverse	356	32	31	16
2% Raf	Forward	208	26	27	18
	Reverse	199	26	28	18
	Forward+Reverse	208	26	27	18

Table S3. Number of genes covered by single molecules of MeSMLR-seq data

Sample	Aligned strand	Number of genes covered by single molecules			
		Maximal	Median	Mean	Standard derivation
2% Glu	Forward	29	4	3	2
	Reverse	24	4	3	2
	Forward+Reverse	29	4	3	2
1% Glu	Forward	22	4	4	2
	Reverse	20	4	4	2
	Forward+Reverse	22	4	4	2
0.5% Glu	Forward	20	4	3	2
	Reverse	20	4	3	2
	Forward+Reverse	20	4	3	2
0.125% Glu	Forward	29	4	3	2
	Reverse	34	4	3	2
	Forward+Reverse	34	4	3	2
2% Gal	Forward	38	3	3	2
	Reverse	40	3	3	2
	Forward+Reverse	40	3	3	2
2% Raf	Forward	26	3	3	2
	Reverse	29	3	3	2
	Forward+Reverse	29	3	3	2

Table S4. Statistics of biological samples and sequencing data generated in this study

Sample	Growth medium			Sequencing data		
	Yeast extract	Peptone	Carbon source	MeSMLR-seq	Bulk-cell RNA-seq	Single-cell RNA-seq
2% Glu	1%	2%	2% Glucose	√	√	√
1% Glu	1%	2%	1% Glucose + 1% Galactose	√	√	
0.5% Glu	1%	2%	0.5% Glucose + 1.5% Galactose	√	√	
0.125% Glu	1%	2%	0.125% Glucose + 1.875% Galactose	√	√	
2% Gal	1%	2%	2% Galactose	√	√	
2% Raf	1%	2%	2% Raffinose	√	√	

Table S6. Statistics of public sequencing data used in this study

Public data	Yeast strain	Growth condition	GEO accession No.	Data format	Reference
**ATAC-seq**	BY4741	YPD	GSE66386 SRR1822155 (rep1) SRR1822156 (rep2)	FASTQ	Schep et al. 2015
**DNase-seq**	W303	YPD	GSE69651 GSM1705337(rep1) GSM1705338(rep2)	CSV	Zhong et al. 2016
**MNase-seq**	BY4741	YPD	GSE61888 SRR1593252(rep1) SRR1593214(rep2) SRR1593251(rep3)	FASTQ	Weiner et al. 2015
**ChIP-seq (Pol2)**	BY4741	YPD	GSE51251 SRR1003615(input) SRR1003615(IP)	FASTQ	Park et al. 2013
**ChIP-exo (Abf1, Cbf1, Mcm1, Rap1 and Reb1)**	BY4741	YPD	GSE93662	GFF	Rossi et al. 2018
**ChEC-seq (Med8 and Med17)**	BY4705	YPD	GSE81289	BED	Grunberg et al. 2016

Table S7. Statistics of bulk-cell RNA-seq data generated in this study

Sample	Biological replicate	Total number of read pairs	Alignment rate (%)
**2% Glu**	Replicate 1	11462015	98.42
	Replicate 2	9091690	98.43
	Replicate 3	8459098	97.56
**1% Glu**	Replicate 1	9066796	98.38
	Replicate 2	10611557	98.29
	Replicate 3	10746015	98.32
**0.5% Glu**	Replicate 1	9691923	97.88
	Replicate 2	10111610	98.33
	Replicate 3	9994920	98.39
**0.125% Glu**	Replicate 1	11210531	98.15
	Replicate 2	9751364	98.20
	Replicate 3	9615422	97.70
**2% Gal**	Replicate 1	9614336	98.16
	Replicate 2	10500154	98.65
	Replicate 3	10784979	98.60
**2% Raf**	Replicate 1	10677473	98.70
	Replicate 2	10395431	98.62
	Replicate 3	9721105	98.50

References for Supplemental materials

Anders S, Huber W. 2010. Differential expression analysis for sequence count data. *Genome Biol* **11**: R106.

Boyle AP, Guinney J, Crawford GE, Furey TS. 2008. F-Seq: a feature density estimator for high-throughput sequence tags. *Bioinformatics* **24**: 2537-2538.

Chen W, Liu Y, Zhu S, Green CD, Wei G, Han JD. 2014. Improved nucleosome-positioning algorithm iNPS for accurate nucleosome positioning from sequencing data. *Nat Commun* **5**: 4909.

Grunberg S, Henikoff S, Hahn S, Zentner GE. 2016. Mediator binding to UASs is broadly uncoupled from transcription and cooperative with TFIID recruitment to promoters. *EMBO J* **35**: 2435-2446.

Hughes AL, Rando OJ. 2014. Mechanisms underlying nucleosome positioning in vivo. *Annu Rev Biophys* **43**: 41-63.

Kim D, Langmead B, Salzberg SL. 2015. HISAT: a fast spliced aligner with low memory requirements. *Nat Methods* **12**: 357-360.

Langmead B, Salzberg SL. 2012. Fast gapped-read alignment with Bowtie 2. *Nat Methods* **9**: 357-359.

Marcel M. 2011. Cutadapt removes adapter sequences from high-throughput sequencing reads. *EMBnetjournal* **17**.

Park D, Lee Y, Bhupindersingh G, Iyer VR. 2013. Widespread misinterpretable ChIP-seq bias in yeast. *PLoS One* **8**: e83506.

Paulo JA, O'Connell JD, Gaun A, Gygi SP. 2015. Proteome-wide quantitative multiplexed profiling of protein expression: carbon-source dependency in Saccharomyces cerevisiae. *Mol Biol Cell* **26**: 4063-4074.

Rossi MJ, Lai WKM, Pugh BF. 2018. Genome-wide determinants of sequence-specific DNA binding of general regulatory factors. *Genome Res* **28**: 497-508.

Schep AN, Buenrostro JD, Denny SK, Schwartz K, Sherlock G, Greenleaf WJ. 2015. Structured nucleosome fingerprints enable high-resolution mapping of chromatin architecture within regulatory regions. *Genome Res* **25**: 1757-1770.

Stegle O, Teichmann SA, Marioni JC. 2015. Computational and analytical challenges in single-cell transcriptomics. *Nat Rev Genet* **16**: 133-145.

Trapnell C, Williams BA, Pertea G, Mortazavi A, Kwan G, van Baren MJ, Salzberg SL, Wold BJ, Pachter L. 2010. Transcript assembly and quantification by RNA-Seq reveals unannotated transcripts and isoform switching during cell differentiation. *Nat Biotechnol* **28**: 511-515.

Weiner A, Hsieh TH, Appleboim A, Chen HV, Rahat A, Amit I, Rando OJ, Friedman N. 2015. High-resolution chromatin dynamics during a yeast stress response. *Mol Cell* **58**: 371-386.

Yuan GC, Liu YJ, Dion MF, Slack MD, Wu LF, Altschuler SJ, Rando OJ. 2005. Genome-scale identification of nucleosome positions in S. cerevisiae. *Science* **309**: 626-630.

Zhang Y, Liu T, Meyer CA, Eeckhoute J, Johnson DS, Bernstein BE, Nusbaum C, Myers RM, Brown M, Li W et al. 2008. Model-based analysis of ChIP-Seq (MACS). *Genome Biol* **9**: R137.

Zhong J, Luo K, Winter PS, Crawford GE, Iversen ES, Hartemink AJ. 2016. Mapping nucleosome positions using DNase-seq. *Genome Res* **26**: 351-364.
